# Assessing the fate and contribution of Foxd1-expressing embryonic precursors and their progeny in palatal development, homeostasis and excisional repair

**DOI:** 10.1038/s41598-024-55486-8

**Published:** 2024-02-29

**Authors:** Georgia Nikoloudaki, Douglas W. Hamilton

**Affiliations:** 1 Department of Anatomy and Cell Biology, Schulich School of Medicine and Dentistry, Western University, London, ON N6A 5C1 Canada; 2 Schulich Dentistry, Schulich School of Medicine and Dentistry, Western University, London, ON N6A 5C1 Canada

**Keywords:** Mesenchymal migration, Differentiation

## Abstract

Oral mucosal tissues heal rapidly with minimal scarring, although palatal mucosa can be associated with excessive fibrosis in response to injury. Investigations on the balance between neovascularization and tissue repair suggests regulation of angiogenesis is an important determinant of repair versus scarring. Associated with pericyte mediated fibrosis in kidney injury, FoxD1 is implicated in growth centres during cranio-facial development, although which cell lineages are derived from these embryonic populations in development and in adult animals is unknown. Using a lineage tracing approach, we assessed the fate of embryonic Foxd1-expressing progenitor cells and their progeny in palatal development and during wound healing in adult mice. During palatal development as well as in post-natal tissues, Foxd1-lineage progeny were associated with the vasculature and the epineurium. Post-injury, de novo expression of FoxD1 was not detectable, although Foxd1-lineage progeny expanded while exhibiting low association with the fibroblast/myofibroblast markers PDGFα, PDGFβ, vimentin, α-smooth muscle actin, as well as the neuronal associated markers S100β and p75NTR. Foxd1-lineage progeny were primarily associated with CD146, CD31, and to a lesser extent CD105, remaining in close proximity to developing neovascular structures. Our findings demonstrate that FoxD1 derived cells are predominantly associated with the palatal vasculature and provide strong evidence that FoxD1 derived cells do not give rise to populations involved directly in the scarring of the palate.

## Introduction

Tissue resident fibroblast populations were originally hypothesized to be a relatively homogeneous cell population in connective tissues, responsible for producing and remodeling the extracellular matrix. However, recent advances in technologies, including lineage tracing, have allowed researchers to identify and investigate subpopulations of fibroblasts, which arise from different lineages of embryonic precursor cells^1–3^. As myofibroblasts are the main source of extracellular matrix (ECM) production during tissue injury^4,5^ and contribute to palatal wound healing where they are considered largely responsible for fibrotic tissue formation^6^, investigation into the cellular origin from which populations originate after injury is important for development of new strategies to improve wound healing outcomes while reducing scar formation^1^.

Potential sources of myofibroblasts progenitors include epithelial cells and endothelial cells, derived through a process termed epithelial-mesenchymal^7^ or endothelial-mesenchymal transition^8^; circulating bone marrow-derived fibrocytes, tissue-resident fibroblasts, and other mesenchymal cells related to blood vessels, including pericytes, adventitial cells, and mesenchymal stem cells (MSCs)^9–12^. Previous research has shown cell progeny derived from Foxd1 embryonic progenitors to be myofibroblast precursors following tissue damage. As a lineage tracing marker, resident Foxd1 lineage-derived populations in the kidney and lung contributed to the myofibroblast populations in induced renal fibrosis following unilateral ureteral obstruction and ischemia reperfusion injury, as well as bleomycin induced lung fibrosis, respectively^13,14^. A recent study from our laboratory using a murine cutaneous wounding model showed that Foxd1-lineage progeny cells expand upon injury and contribute to the stromal population and α-SMA-positive myofibroblasts, but did not contribute to perivascular and endothelial cells^15^. These studies do show that Foxd1 lineage derived populations can contribute to myofibroblast precursors following tissue injury, but the cell of origin varies depending on the tissue.

To date, evidence of perivascular cell recruitment and differentiation into myofibroblasts appears to be organ specific^3,16^, and, currently, whether this process occurs within the palatal mucoperiosteum has yet to be tested. Here, a lineage tracing approach was used to trace populations of cells derived from embryonic Foxd1-expressing progenitors during development, homeostasis, and excisional wound healing. Our data indicate that in the palate, FoxD1-expressing embryonic cells contribute to palatal formation, but Foxd1-lineage progeny primarily associate with blood vessels during embryonic development as well as in adult tissues. After injury Foxd1-lineage progeny expand but remain associated with neovascular structures and do not contribute to myofibroblast populations associated with palatal scarring. This works provides further evidence that the role of tissue resident cell populations involved in angiogenesis and fibrosis can be identified in adult organisms based on specific embryonic markers.

## Results

### Foxd1-lineage-positive (FLP) cells and their progeny are present at the developing secondary palate during embryogenesis

**Figure 1:**
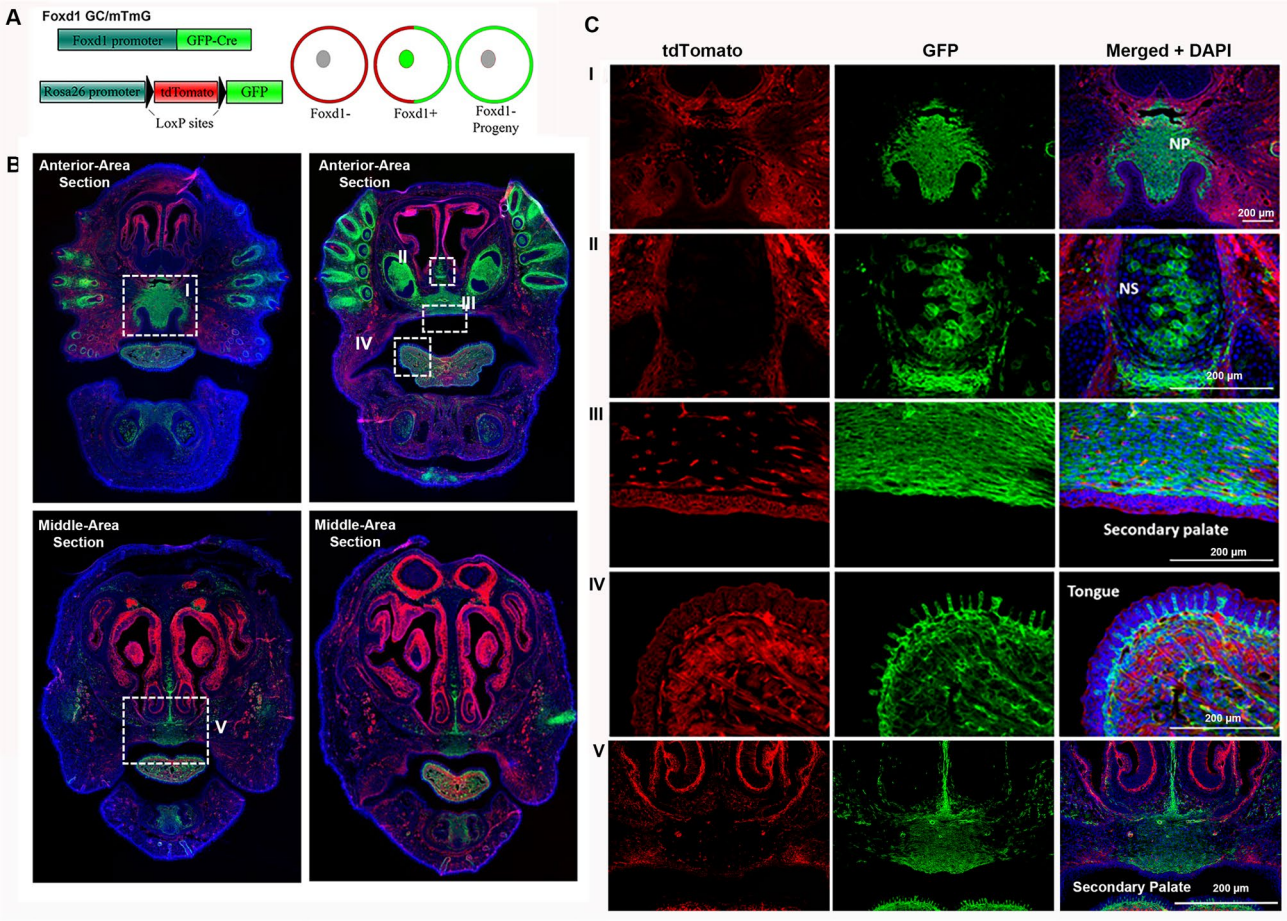
Foxd1-positive embryonic lineages in the developing embryos contribute to FLP cell populations within the developing orofacial tissues and palate. (**A**) *Foxd1GC* mice were crossed with an *mTmG* reporter to investigate FLP (Green) and FLN Foxd1-lineage negative (Red) contributions to palatal and orofacial tissues during embryonic development. (**B**) Embryonic tissues were harvested at E17.5 and sectioned in the frontal plane to include the developing palatal tissues from the anterior to the most posterior part of the palate. FLP cells are present within the head, particularly at the anterior area. FLP cells are not present in the most posterior area palate. Representative images from at least 3 embryos are shown. The areas highlighted in the white rectangle are magnified in the panels i-v in panel C. (**C**) Magnified areas *NP* nasal process, *P* palate, *NS* nasal septum, *T* Tongue. Scale bar: 200 μm.

Using a lineage tracing strategy to track Foxd1 derived populations (Fig. 1A, Supplemental Fig. 1A), we investigated the contribution of this lineage to cell populations during early maxillofacial development using Foxd1 GC/mTmG mice. At E17.5, Foxd1-lineage-positive (FLP) cells were present within the developing head, particularly at the area of the primary palate (median palatine process and nasal process) (Fig. 1B). Highest expression of FoxD1 was evident in the anterior aspect of the palate, with declining expression evident in a posterior direction (Fig. 1B). Within the developing orofacial tissues, FLP cells contributed to various mesenchymal and ectomesenchymal tissues. FLP cells were associated with the mesenchyme within meninges, the nasal process of the secondary palate and the tongue (Fig. 1B, Ci, ii, iii, iv, v). Similar results were evident in Foxd1 GC/Ail4 reporter mice, which showed the presence of FoxD1-positive cells in the nasal process and palatal shelve at E12.5 (Supplemental Fig. 1). By day 17.5, FoxD1-lineage progeny predominantly colonized the secondary palate, with fewer FoxD1-expressing cells evident (Supplemental Fig. 1).

### Foxd1-lineage-positive (FLP) cells and their progeny contribute to vascular cell populations within the developing secondary palate

**Figure 2:**
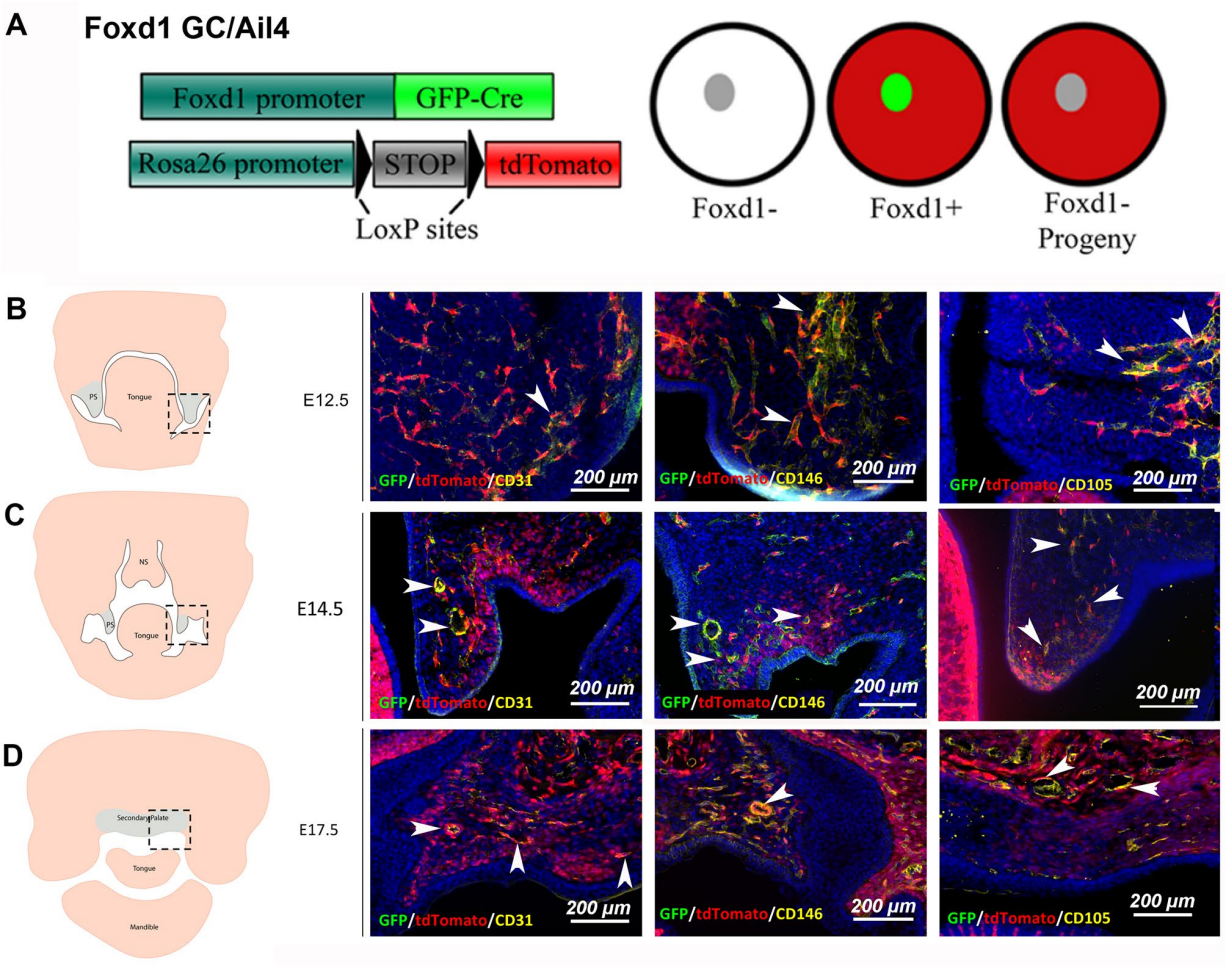
Foxd1-positive progenitor cells and their progeny contribute to vascular cell populations within the developing secondary palate. (**A**) To characterize the differentiated populations represented by the Foxd1 lineage, the constitutively active Foxd1GC line crossed to the Ai14 reporter was assessed. Tissues were counterstained for CD31, CD146 and CD105 to detect developing vasculature within the palatal tissues from E12.5 to E17.5. Scale bar: 200 μm. (**B**–**D**) Schematic representation of murine orofacial tissues at E12.5–E17.5 are shown at each stage, highlighting the regions (black dashed box) that contribute to secondary palate development (palatal processes/shelves). Scale bar: 200 μm. White arrowheads indicate areas with overlapping signal. *PS* palatal shelve, *NS* nasal septum. Separate channel images are shown in supplemental file 2.

**Figure 3:**
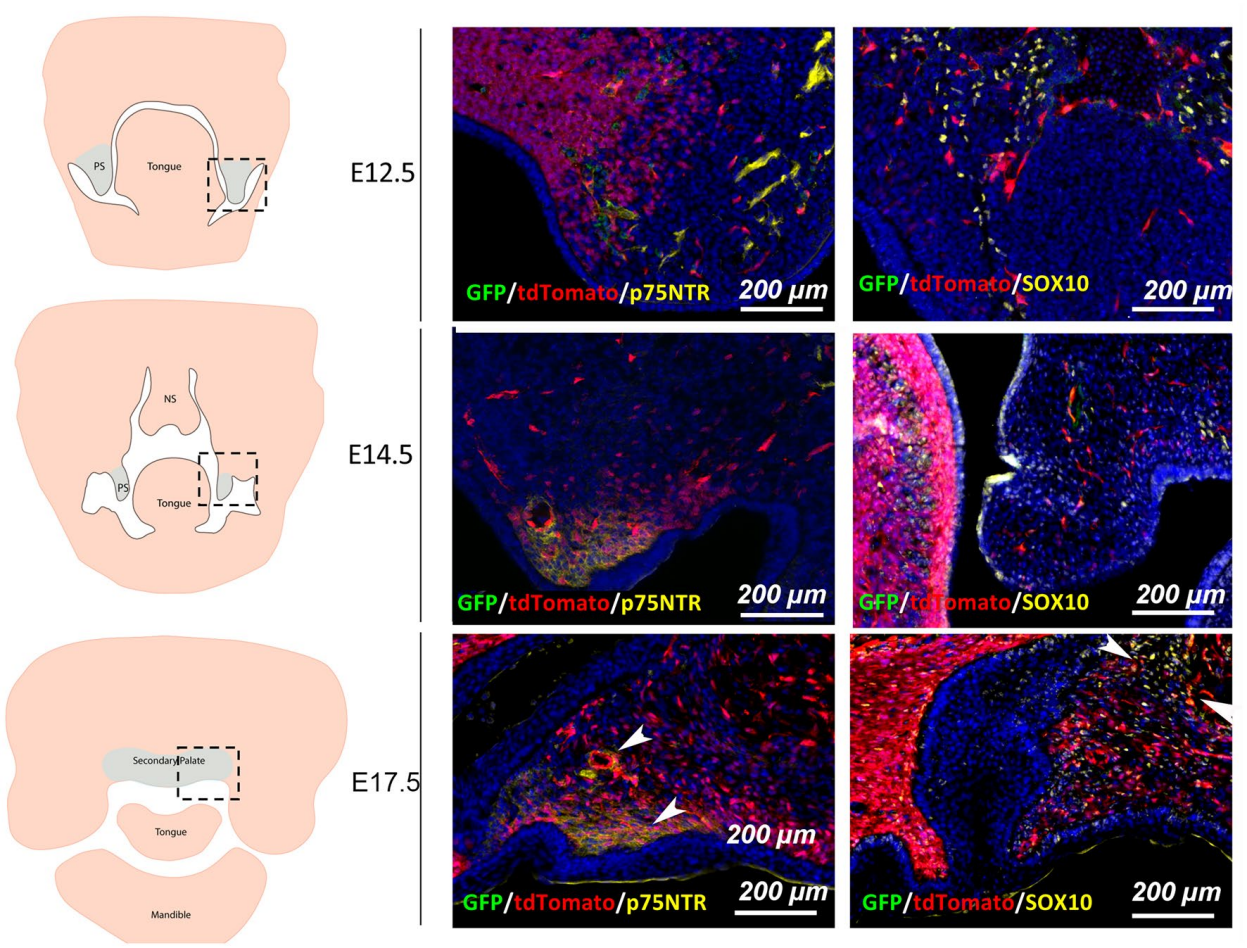
From E12.5 to E17.5, FLP cells (red) do not express markers associated with early neural crest-derived cell populations. Tissues were stained for p75NTR and SOX10 to detect whether FLP cells within the palatal tissues at E12.5 to E17.5 continued to express markers associated with migratory neural crest cells (< E10.5). Scale bar: 200 μm. White arrowheads indicate areas with overlapping signal.

To determine the type of cells that are FLP during the various stages of the palatal embryonic development, tissues from embryos heterozygote for both Foxd1GC and Ai14 alleles were labelled for cell markers associated with vasculature structures and neural crest cells. High magnification images were taken of tissues labelled for CD31, CD146 and CD105 in tissues at E12.5 (Fig. 2A) E14.5 (Fig. 2B) and E17.5 (Fig. 2C). Single channel images are shown in supplemental file 2. At all timepoints, tdTomato positive cells and their progeny (FLP) exhibited considerable overlap with all tested vascular markers. Increased overlap of all markers was noted with increasing time. However, Foxd1-lineage-negative (FLN) cells were also noted to label for these markers. By E12.5 of embryogenesis, tdTomato-positive cells showed minimal overlap with SOX10 and p75NTR neural crest markers, indicating that Foxd1-lineage positive cells do not express either of these markers at this stage of development (Fig. 3).

### Foxd1-expressing cells are rare in postnatal tissue during homeostasis and following wounding

**Figure 4:**
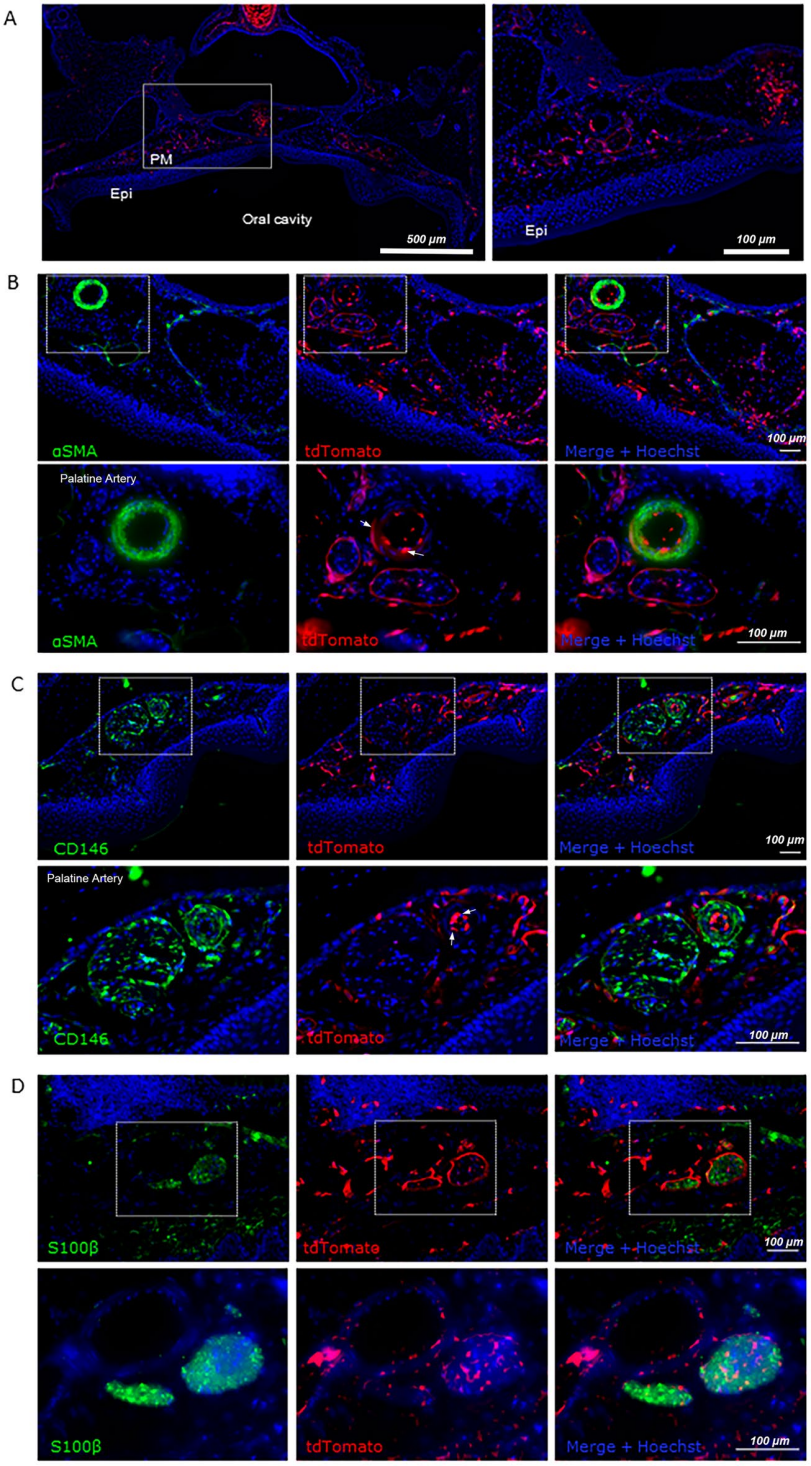
Foxd1-positive progenitors and their progeny give rise to a subset of perivascular and perineural populations within the quiescent adult palatal mucoperiosteum. To characterize the differentiated populations represented by the Foxd1 lineage, the constitutively active Foxd1GC line crossed to the Ai14 reporter was assessed. (**A**) Within the unwounded palatal mucoperiosteum Foxd1 expressing (green) cells were absent. Progeny of FoxD1 expressing embryonic precursors were sparse in the unwounded palatal mucoperiosteum and mainly associated with blood vessels and the perineurium of nerves. The area highlighted in the white rectangle is magnified on the below. Adult mouse palatal tissues were labeled for (**B**) smooth muscle and myofibroblast marker αSMA, showing overlap in the palatine artery (White arrows). The area highlighted in the white rectangle is magnified below. Co-localization of tdtomato with the endothelial and pericyte marker (**C**) CD146 showed limited overlap between the signals (White arrows). The area highlighted in the white rectangle is magnified on the below (**D**) S100β, a marker for Schwann cells demonstrated that FoxD1 progeny localized near the epineneurium and perineurium. The area highlighted in the white rectangle is magnified on the below. *PM* palatal mucoperiosteum, *Epi* oral epithelium, Scale bar panel A left: 500 μm, A right and B–D: 100 µm.

To determine whether Foxd1 is expressed in normal adult palatal mucoperiosteum, palatal tissues were harvested from heterozygote Foxd1GC/Ai14 mice. Recombination in Foxd1-Cre-GFP is constitutively active in cells expressing the Foxd1 promoter, inducing expression of tdTomato driven by the ubiquitously expressed Rosa26 promoter. Foxd1 expression was not observed within the healthy/unwounded palatal samples taken, as indicated by the absence of GFP/Foxd1 signal (Fig. 4A). In the unwounded palatal mucoperiosteum, tdTomato signal was observed in some cells that were mainly associated with blood vessels and the perineurium of nerves, suggesting that these cells are derived from Foxd1 positive precursors during development [Foxd1-lineage-positive cells (FLP cells)] (Fig. 4A). To determine more specifically which populations these FLP cells were contributing to within the palatal tissues, adult mouse palate was labeled with several markers including the smooth muscle and myofibroblast marker α-SMA (Fig. 4B), the endothelial/pericyte marker CD146 (Fig. 4C), and S100β to identify Schwann cells (Fig. 4D). Immunolabelling with α-SMA, CD146 and S100β showed that FLP cells are in close association with the microcirculation, where they give rise to certain populations of mural cells and endothelial cells. FLP cells also present in the perineurium of nerves as well as some cell population within the endoneurium (endoneural fibroblasts/pericytes).

### Foxd1-expressing cells are absent in postnatal palatal mucoperiosteum following injury

To identify perivascular cell activation within wounded palatal tissue, two different mouse strains were utilized. Both *B6;129S4-Foxd1tm1(GFP/cre)Amc/J,* and the inducible *B6;129S4-Foxd1tm2(GFP/cre/ERT2)Amc/J* strains were bred with *B6.CgGt(ROSA)26Sortm14 (CAG-tdTomato)Hze/J.* Non-inducible mice containing both the Foxd1 driven, Cre-GFP fusion protein, and the Rosa26 driven, tdTomato preceded by a loxp-flanked stop codon, express GFP in any cells actively expressing Foxd1, and tdTomato in any cell derived from a Foxd1 positive precursor cell (Foxd1-*lineage*-positive cells; FLP cells). The inducible strain adds the ability to temporally control when the Cre recombinase is functional through the injection of tamoxifen. Wound repair was assessed following excisional wounding in the palatal mucoperiosteum of these mice and histological analysis was performed to investigate the contribution of Foxd1 positive cells and their progeny in wound healing. Masson's trichrome images at day 6 and 10 are shown in supplemental Fig. 3 to show the area of wounding (Figure S3A), as well as the granulation tissue and wound appearance. In summary, re-epithelialization is complete by day 6 (Figure S3B), with extensive granulation tissue developing underneath. However, even at day 10 post-injury, the granulation tissue has very low collagen content (Figure S3C).

**Figure 5:**
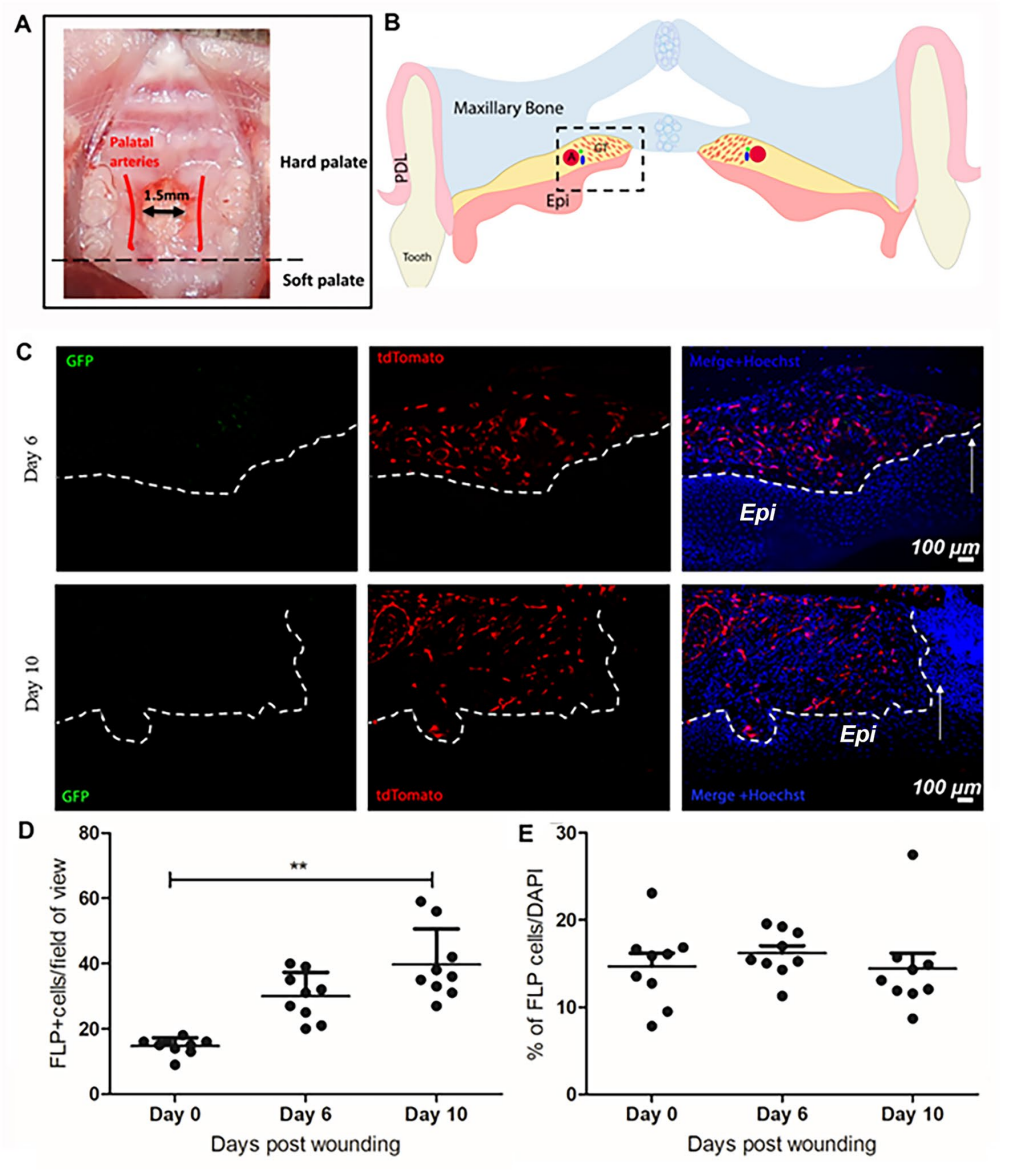
Foxd1-expressing cells are absent in postnatal palatal mucoperiosteum following injury. (**A**) Image showing the excisional wound created in the palatal mucoperiosteum between the palatine arteries. (**B**) Schematic representation: Coronal view of palatal wounded tissues. The area of interest is highlighted in black dashed box (*Epi* oral Epithelium, *PDL* periodontal ligament, *GT* granulation tissue, *A* major palatine artery). (**C**) Anti-GFP antibody was used to detect GFP signal in the wounded tissues. Absence of signal verified that GFP (Foxd1) is not expressed in adult tissues upon injury. White arrows in merged panel indicate the leading edge of the wound. White dashed line indicates the border between the epithelium (Epi) and the granulation tissue. (**D**) Quantification analysis of the tdTomato-positive cells present within the wounded tissues revealed that FLP cells appear at 6 days post wounding within the granulation tissues and increase in density over time to 10 days post wounding. Quantification presented as percentage of FLP cells/field of view. (**E**) Quantification presented as percentage of FLP cells/total cells (tdTomato/Hoechst). Arrows point to the boundary of healthy tissue with the edge of the excisional wound. Scale bar: 100 μm.

To determine if Foxd1-expressing cells or their progeny participate, or are activated, in palatal wound healing, full-thickness excisional wounds were created in the palates of constitutively active *Foxd1*GC/*Ai14* mice (Fig. 5A, B). Tissues were harvested at days 6 and 10 post-wounding and the tissues were subjected to histological analysis (N = 3 animals/time-point). Upon wounding, GFP-positive cells were absent from the granulation tissue, confirmed by the absence of signal of anti-GFP at days 6 and 10 post-wounding (Fig. 5C). tdTomato-positive cells (FLP cells) were present at 6 days post wounding within the granulation tissues and increased in density by 10 days post wounding (Fig. 5D, E). To quantitatively determine if postnatal expression of Foxd1 was contributing to the FLP populations observed in these tissues, recombination in a tamoxifen inducible Cre model was investigated by crossing the *Foxd1GCE* line with the *Ai14* reporter. Tamoxifen was delivered to postnatal mice after wounding and tissues were harvested for histological assessment of recombination at days 6 and 10 post-wounding. Histological images revealed the absence of recombination, as indicated by the lack of cells expressing tdTomato (N = 2) (Supplemental Fig. 4). Combined with the expression patterns observed during embryogenesis, these data suggest that the FLP cells present within the palatal mucoperiosteum were primarily derived from embryonic precursors that expressed *Foxd1*, and that de novo expression of *Foxd1* in postnatal palate during homeostasis and wound healing was absent.

### Foxd1-lineage-positive cells contribute to diverse populations in adult palatal mucoperiosteum upon injury

**Figure 6:**
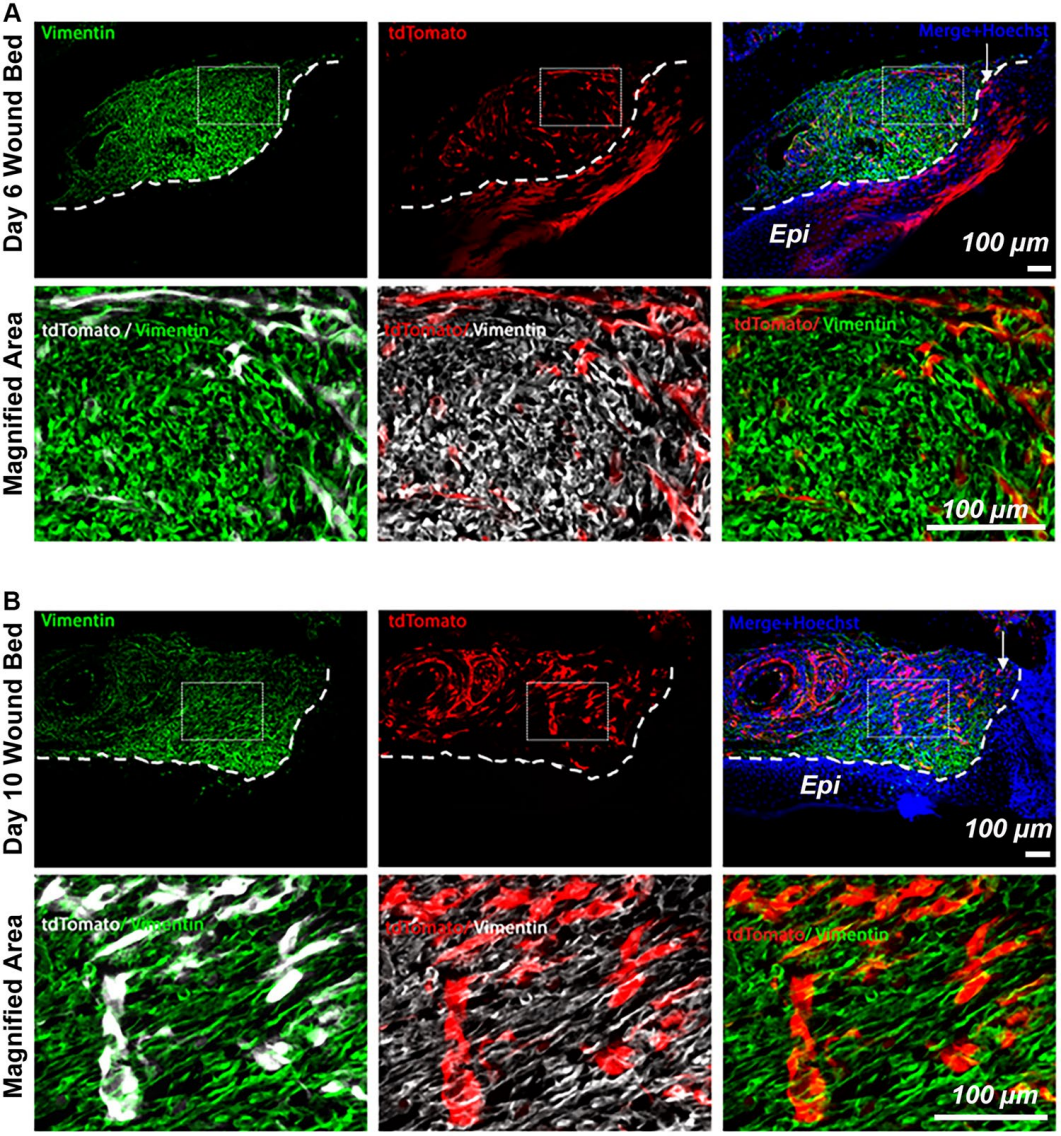
FLP cells within day-6 and -10 granulation tissue are not a prominent Vimentin-positive cell population. FLP cells (tdTomato positive) show minimal overlap with staining for Vimentin (green) within the granulation tissue. Nuclei are stained with Hoechst dye (blue). Representative images are shown from a sample of 3 mice. White arrows indicate the leading edge of the wound. White dashed lines indicate the border between the epithelium (Epi) and the granulation tissue. Scale bar: 100 μm.

**Figure 7:**
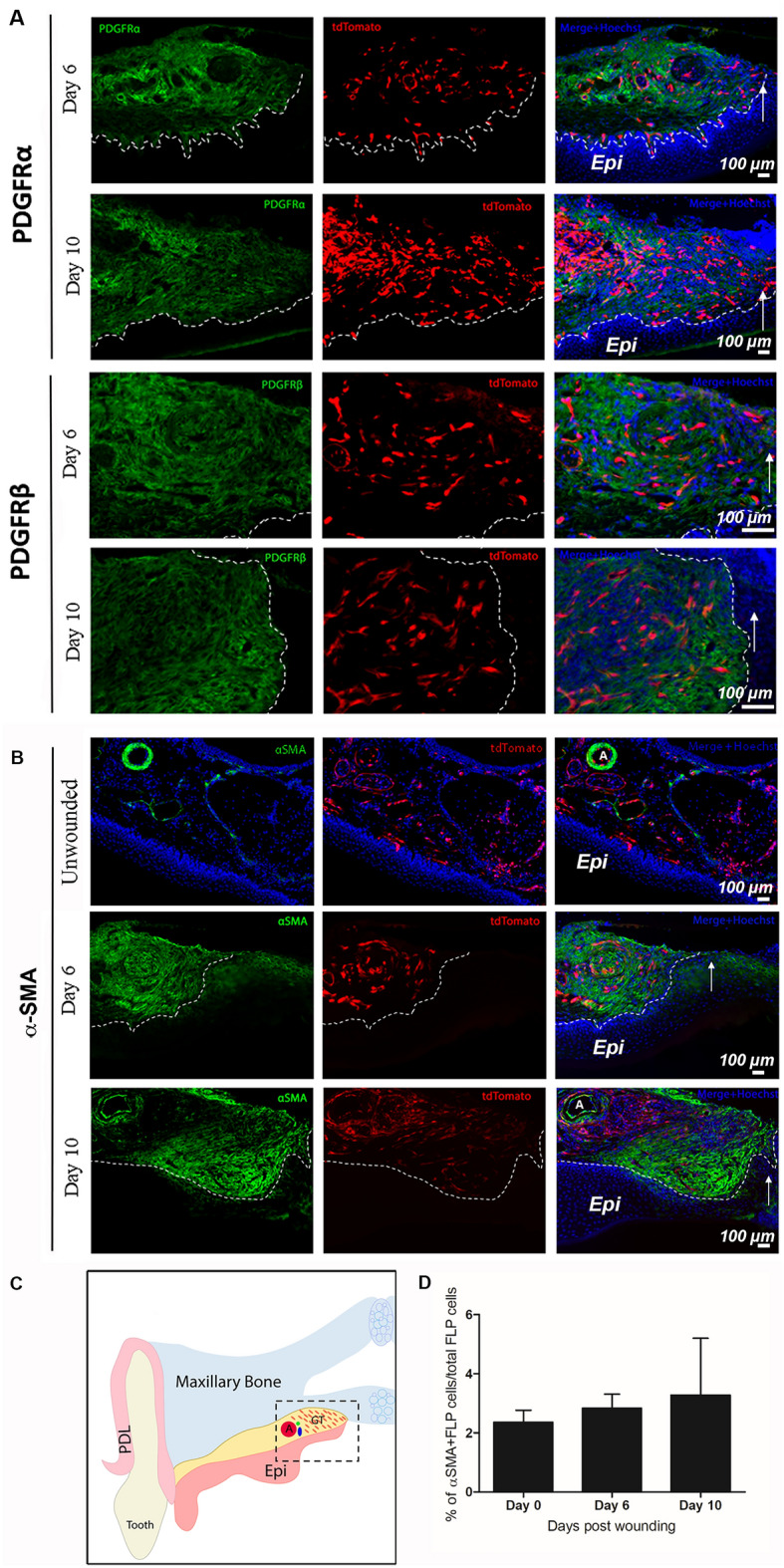
FLP cells within day-6 and -10 granulation tissue are not primarily associated with fibroblast markers. (**A**) FLP cells (tdTomato positive) show minimal overlap with staining for fibroblast markers PDGFRα and PDGFRβ (green) within the granulation tissue. Nuclei are stained with Hoechst dye (blue). Representative images are shown from a sample of 3 mice per timepoint. Arrows indicate leading wound edge. White dashed lines indicate the border between the epithelium and the granulation tissue. (**B**) Foxd1-lineage-positive cells begin to appear at 6 days post wounding at the wound edge and increase in density over time to 10 days post wounding. These cells do not contribute significantly to the myofibroblast populations as shown by the minimal overlap between tdTomato and αSMA signals. White arrows indicate the leading edge of the wound. White dashed lines indicate the border between the epithelium and the granulation tissue. (**C**) Schematic representation: Coronal view of palatal wounded tissues. The area of interest is highlighted in black dashed box (*Epi* oral epithelium, *PDL* periodontal ligament, *GT* granulation tissue, *A* major palatine artery). (**D**) Quantification presented as percentage of αSMA-positive FLP cells/total FLP cells showed that only minimal fraction of FLP cells contribute to the myofibroblast population (unwounded < 3%; day 6 < 3%; day 10 < 4%, N = 3 animals/timepoint). Scale bar = 100 µm.

To determine more specifically which populations these cells were contributing to, adult mouse wounded palatal tissues from *Foxd1*GC/*Ai14* mice were labeled with smooth muscle and myofibroblast marker α-SMA, several markers expressed in fibroblasts (vimentin, PDGFRα, PDGFRβ), the endothelial marker CD31, the pericyte marker CD146, as well as the schwann cell associated markers p75NTR and S100β. The FLP cells within the wound tissue were present in the granulation tissue but did not show any overlap with vimentin at either day 6 or day 10 post-wounding (Fig. 6A, B), or PDGFRα, PDGFRβ at either day 6 or day 10 post-wounding (Fig. 7A). Quantification of the α-SMA-positive FLP cells showed that only a small fraction of FLP cells contribute to the total myofibroblast population (unwounded < 3%; day 6 < 3%; day 10 < 4%, N = 3 animals/timepoint) (Fig. 7C, D).

**Figure 8:**
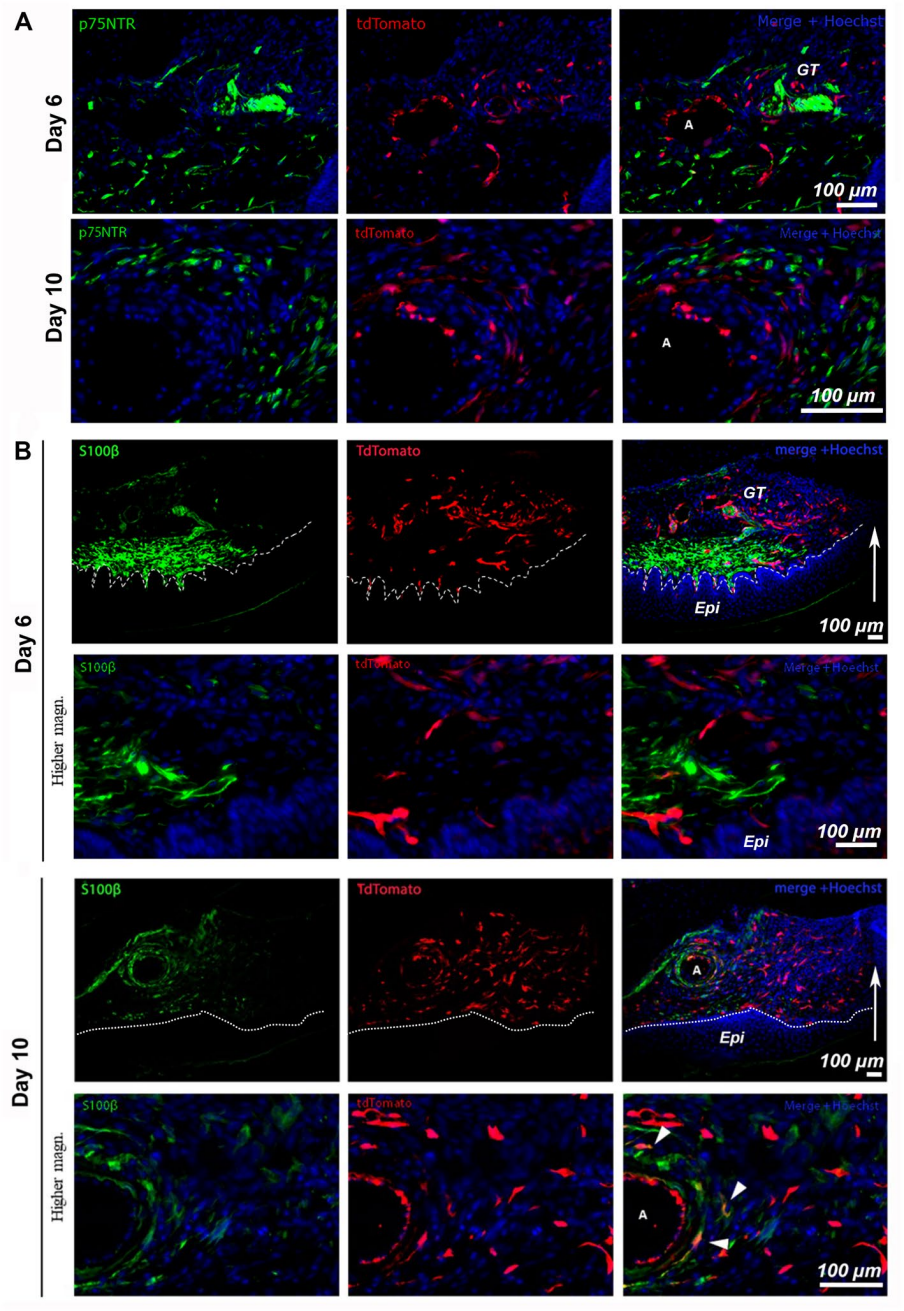
FLP cells are minimally associated with p75NTR- or S100β expressing populations distal to the wound. Immunofluorescent staining for p75NTR (green) (**A**), day 6 and day 10 post wounding, did not reveal any significant overlap between regions of tdTomato-positive cells with p75NTR suggesting that FLP cells do not actively express this marker in repair. (**B**) Immunofluorescent staining for S100β (green) at day 6 and 10 post wounding. White arrows indicate the leading edge of the wound. White dashed lines indicate the border between the epithelium (Epi) and the granulation tissue (GT). White arrowheads indicate FLP cells positive for S100β marker, as indicated by the colocalization of the signal. N = 3 animals/timepoint. Scale bar: 100 μm.

To assess overlap of FLP cells with cells of neuronal origin, wounded tissue was stained at day 6 and 10 post-wounding for p75NTR (Fig. 8A). No overlap between FLP cells and p75NTR was observed. To determine whether FLP cells contribute to any of the emerging populations of cells that colonized the granulation tissue, the Schwann cell marker S100β was used. The histological analysis revealed that S100β-positive cells are largely absent within the granulation tissue (Fig. 8B). Quantification analysis of S100β-positive FLP cells distal to the wound, in close proximity to the neurovascular bundle of the palate, showed that S100-positive FLP cells constitute only a minor part of the FLP population in normal tissue and at early stages of wound healing-day 6 (< 3%, N = 3 animals/timepoint), while their density increased by day 10 (9%, N = 3 animals/timepoint).

### Foxd1 lineage progeny contribute to vascular cell populations in wound repair

**Figure 9:**
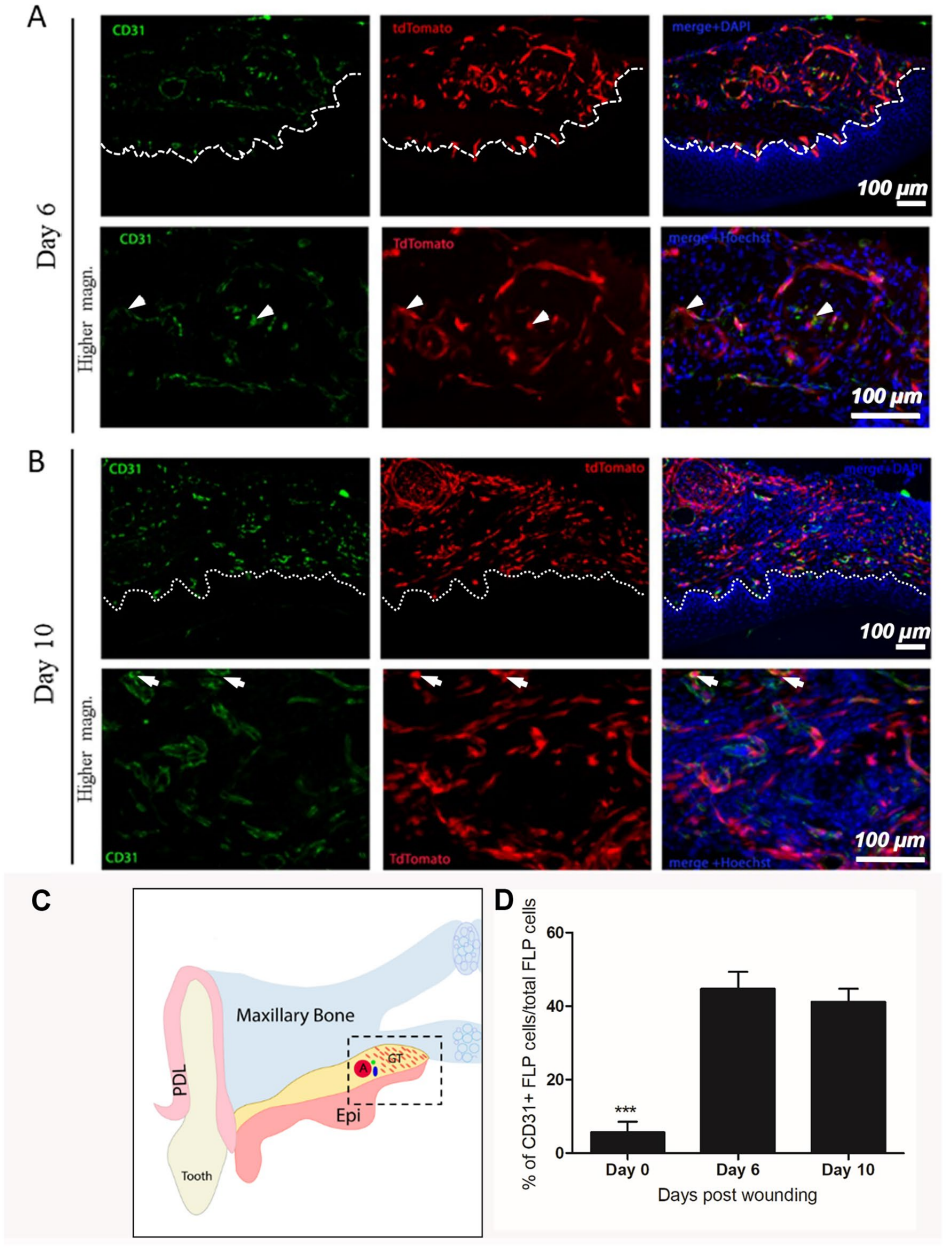
FLP cells within day-6 and -10 granulation tissue contribute to CD31-positive populations within the wounded tissues. Immunofluorescent staining for CD31 (green) at days 6 (**A**) and 10 (**B**) post-wounding. White dashed lines indicate the border between the epithelium (Epi) and the granulation tissue (GT). (**C**) Schematic representation: Coronal view of palatal wounded tissues. The area of interest is highlighted in black dashed box (*Epi* oral epithelium, *PDL* periodontal ligament, *GT* granulation tissue, *A* major palatine artery). (**D**) Quantification presented as percentage of CD31-positive FLP cells/total FLP cells. Data were analyzed using 1-way ANOVA and Tukey post hoc test. N = 3 (***P < 0.001). Scale bar: 100 μm.

**Figure 10:**
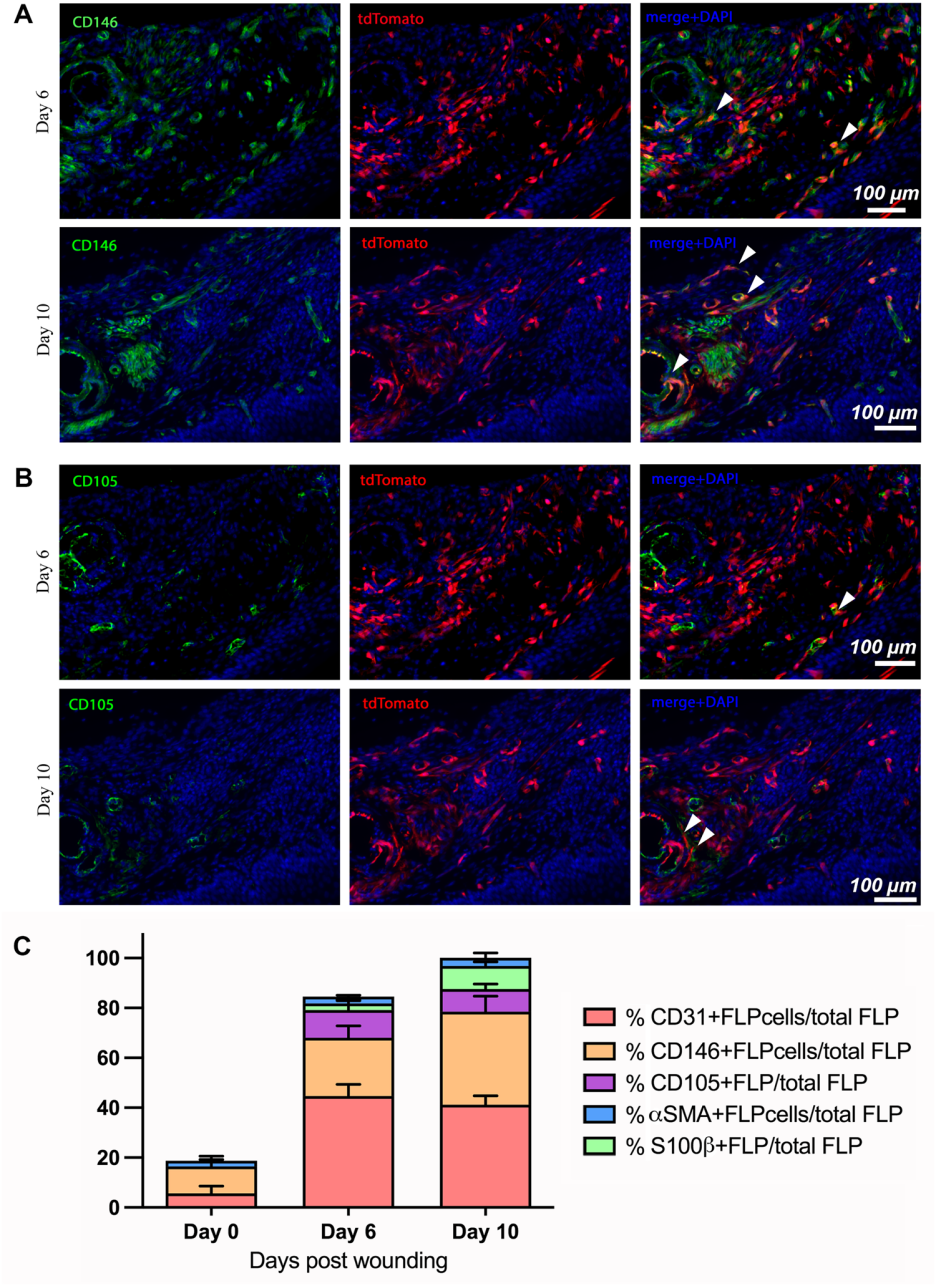
FLP cells within day-6 and -10 granulation tissue contribute to CD146-positive and CD105-positive populations within the wounded tissues. (**A**) Immunofluorescent staining for CD146 (green) at days 6 and 10 post-wounding. White arrowheads indicate representative cells with dual labeling between FLP cells and CD146-positive cells (**B**) Immunofluorescent staining for CD105 (green) at days 6 and 10 post-wounding. White arrowheads indicate representative cells with dual labeling between FLP cells and CD105-positive cells. (**C**) Overall contribution and expression profile of FLP cells. Quantification presented as percentage of selected-marker-positive FLP cells/total FLP cells at normal and wounded palatal tissues at days 6 and 10 post-wounding. Scale bar: 100 μm.

To determine which populations FoxD1 lineage progeny were, adult mouse wounded palatal tissues were labeled with markers associated with vascular cells (endothelial cells, pericytes). In unwounded adult tissues less than 3% of FLP cells were positive for CD31 marker (*Data not shown*). Interestingly, post-wounding a significant overlap was evident between tdTomato (FLP cells) and CD31, suggesting that FLP cells activate and significantly contribute to the increasing endothelial cell population post-wounding (> 40% of FLP cells were found to be CD31 positive) (Fig. 9A, B). However, it was evident that the FLP population only give rise to a subset of these populations, with FLN cells also contributing. Similar observations were made when antibodies to CD146 were utilized, with less tdTomato overlap with CD105 which labeled endothelium particularly at day 10 (Fig. 10A, B). A profile of expression markers with FLP cells demonstrated that cells were predominantly associated with CD146 and CD31 post-injury (Fig. 10C).

## Discussion

Using a lineage tracing approach, embryonic progenitors that differentially expressed Foxd1 were investigated in a murine model. In this study we identified that Foxd1-lineage progeny expanded after injury and contributed to the formation of neovascular structures. Interestingly, the Foxd1-lineage progeny populations described here do not directly overlap with distinct embryonic lineages that have been previously identified within different organs in murine models, such as in skin, kidney, lung, liver^13–15,17,18^.

Evidence suggests that identification of fibroblast lineages could be used to a degree as a predictor of cell function, but that this is likely only true within a specific tissue location. In fact, lineage itself may simply predict a cell's location, which in turn defines its phenotype^15^. In the palatal mucoperiosteum palatal soft tissue is a rigid mucoperiosteum; mucosa and the periosteum are merged and tightly attached to the palatal bone, leading to excessive scarring upon wounding^19^. Here, we explored whether binary expression of *Foxd1* during embryogenesis could be used as a new potential marker of fibroblasts within the oral cavity and the repair process in the palatal mucoperiosteum, and to compare our findings with evidence from murine skin healing. Our data shows by day E12.5, FLP cells do not express certain neural crest stem cell associated markers (p75NTR and Sox10) but the downregulation of these genes at this stage of embryogenesis has been described^20^ and not all neural crest stem cells express these markers. However, we do show here that FLP cells represent a major contributor of pericytes and endothelial cells during the neovascularization of the developing palatal tissues. Additionally our results show that Foxd1-lineage positive cells contribute to various mesenchymal and ectomesenchymal tissues within the developing orofacial tissues, such as mesenchyme within meninges, nasal process of the secondary palate, whisker follicles, nasal cartilage, cells within the dental mesenchyme, the dental papilla and dental follicle of developing teeth, the eye retina and fiber bundles in the extraocular muscles, and the tongue, all of which are of neural crest- or mesodermal origin. More detailed analysis of this cell population revealed that Foxd1-lineage positive cells were also sparsely distributed in lateral maxillary processes contributing to cells of the neovasculature. It is of interest that angioblasts are the only cells of mesodermal origin so far identified that move into the stream of migrating neural crest cells^21^, apparently as soon as the neural crest cells have formed as a mesenchymal population lateral and rostral to the mesodermal mesenchyme. This early incursion of mesodermal angioblasts into an apparently otherwise pure neural crest cell population enables blood vessels to form equally quickly within the neural crest-derived and mesoderm-derived mesenchyme of the head^21^. Evidence from lineage tracing studies suggest that in the craniofacial region of mouse embryos the endothelial cells of blood vessels are of mesodermal origin and the muscular components of the vessel walls (pericytes) are derived from neural crest^21^.

Overall, limited information or analysis of cell populations through lineage tracing experiments has been performed in the oral cavity or the hard palate. Utilizing lineage tracing techniques in *Wnt1-Cre;Zsgreen*^*fl/fl*^ mice it was demonstrated that all the tendons and mesenchyme embedding the soft palate muscles are neural crest-derived, proposing that the posterior attachment of the soft palate to the pharyngeal wall is an interface between the neural crest- and mesoderm-derived mesenchyme in the craniofacial region^22^. Although regional clonal diversity was also recently demonstrated in the cells of the epithelium that covers the hard palate in mice^23–25^, there is limited analysis of the stromal cells of the palatal mucoperiosteum. Lineage tracing experiments have identified that neural crest-derived cells persist in adult tissues in some areas of the oral mucosa and that Lgr51-lineage-positive cells participate in the maintenance of the stroma^26^. Our findings provide further evidence about the potential origin of the vasculature structures within the developing palate.

In the adult mouse, the cell populations arising from Foxd1-lineage positive cells contributed to a relatively small proportion of cells in the quiescent adult palatal mucoperiosteum, mainly associated with blood vessels, where they give rise to some smooth muscle cells of peripheral veni, pericytes and some endothelial cells. FLP cells also constituted to cells of the perineurium of nerves as well as some cell populations within the endoneurium (endoneural fibroblasts/pericytes). However, our data shows that upon experimental wounding this population is activated contributing to cells mainly associated with neovascularization of the wound. While CD31^+^FLP cells were sparse in the unwounded tissues, upon wounding more than 40% of FLP cells were CD31^+^. In contrast, no significant overlap with any fibroblast or myofibroblast markers was identified. This finding is in direct contrast with evidence of the Foxd1-lineage positive cell population in dermal wound healing. In skin the Foxd1 lineage represented 50–70% of the dermal fibroblasts and contributed to myofibroblast progenitors, and only minimally to vascular cells^15,27^, providing further evidence about differences between oral and skin healing characteristics. Foxd1-derived populations in skin have also been shown to display enhanced fibrogenic potential compared to their lineage-negative counterparts, as shown by FACS sorting, gene expression and functionality assays^15^.

Collectively, these findings providing further evidence about differences between oral and skin healing characteristics, suggesting that even though this population has a common embryonic signature, the functional properties of its terminally differentiated progeny is largely dependent on the physico-mechanical characteristics of the mature tissue: in skin, a tissue prone to scarring, this population accounts for almost 50% of the fibroblasts, while in the palate, a tissue that does not typically scar (unless major cleft-palate reconstruction surgery is performed), Foxd1 progeny primarily gives rise to vasculature-related cells advancing the healing outcome via neovascularization of the granulation tissue. Revascularization of the wound is essential for the repair and regeneration of the tissue. Functional and persistent neoangiogenic activity has been shown to result in favorable wound healing outcomes across different tissues, such as the myocardium^28^ and skin^29^. This evidence of the distinct roles of Foxd1 progeny in skin and palatal tissues could be considered a contributing factor of the oral mucosa to heal without scarring.

In the present study, Foxd1 expression by embryonic progenitors enabled the tracking of distinct cells that contribute to the vasculature of the developing palatal tissues and that maintained their spatial differences into adulthood and regeneration. Our findings further support that cell populations across different tissues, although originating from common embryonic lineages, they present different phenotypes in mature and wounded tissues. This observation points to the significance of cell-extrinsic factors (extracellular environment, from tissue-specificity perspective, ECM composition and microenvironment biomechanical properties) as determinants of cell fate and behaviour across different tissues, and that could further explain the different healing patterns among them. Furthermore, our findings could lead to the development of model system that allows separation of vascular entities and pro-fibrotic myofibroblast behaviors in the same healing model. Since poor wound healing and scarring are associated with reduced vascularization and vascular stability, this model could be utilized to assess changes in vascular/fibrotic balance, and the final healing outcome.

## Experimental procedures

### Resource availability

Further information and requests for resources and reagents should be directed to and will be fulfilled by the corresponding author, Douglas W. Hamilton (dhamil2@uwo.ca). Materials Availability: This study did not generate new unique reagents. All unique/stable reagents generated in this study are available from the lead contact without restriction. All data and archive of the unprocessed data are available from the lead contact without restriction.

### Mice

All animal procedures were performed in accordance with protocols approved by the University Council on Animal Care at The University of Western Ontario and in strict adherence to Canadian Council on Animal Care guidelines. *Foxd1GC* (Foxd1tm1(GFP/Cre)Amc/J), *Foxd1GCE* (B6;129S4-Foxd1tm2(GFP/Cre/ERT2)Amc/J), *Ai14* (B6.Cg-Gt(ROSA)26Sortm14(CAG-tdTomato)Hze/J), *mTmG* (B6.129(Cg)-Gt(ROSA)26Sortm4(ACTB-tdTomato,-EGFP)Luo/J) mice were purchased from The Jackson Laboratory (Farmington, CT). Schematics and brief descriptions of each of the Cre/Lox models used in this study are provided in Figs. 1 and 2. Mice were genotyped as per The Jackson Laboratory’s instructions. Mice that were heterozygous for the alleles of interest were used for all experiments.

### Murine embryonic tissues

*Foxd1-Cre-GFP* mice were bred with *ROSA26-mTmG/þ* mice to produce offspring that express a Cre-GFP fusion protein under control of the Foxd1 promoter. The reporter used was *mT/mG* (membrane-Tomato before Cre/membrane-GFP after Cre) expressed from the Rosa26 locus. In bigenic mice, cells that expressed Foxd1 at some point in development are green and cells that never expressed Foxd1 are red. Embryonic tissues were harvested at E17.5 and sectioned in the frontal plane to include the developing palatal tissues from the anterior (hard palate) to the most posterior (soft palate) part of the palate. For timed pregnancy, embryos heterozygous for the alleles *Foxd1GC/ Ai14* were used. Females were monitored for vaginal plugs and the day following was considered embryonic day 0.5 (E0.5). Embryos were isolated from pregnant females at E12.5, E14.5, and E17.5. For all time-points, at least 3 embryos were analyzed from at least 2 independent litters.

### Palatal wounds

For experiments, mice heterozygous for the alleles *Foxd1GC/Ai14*, and *Foxd1GCE/Ai14* (20 weeks of age) were anesthetized with an intraperitoneal injection of buprenorphine (0.05 mg/kg), followed by an injection of ketamine (90 mg/kg) and xylazine (5 mg/kg). One full-thickness excisional wound was made with a 1.5 mm disposable biopsy punch (Integra™ Miltex®, Integra York PA, Inc.) on the hard palate. The localization of the palatal punch biopsy was standardized with the anterior edge of the wound to be aligned with the first molar^30^ (Fig. 6A, B) to avoid traumatizing the palatal arteries which run on either side of the wound. The animals received 0.05 mg/kg Buprenorphine by subcutaneous injection twice daily for 48 h post-surgery as an analgesic. Animals were maintained on a standard lab chow powdered food diet and were allowed food and water ad libitum for the duration of the experiment. Excised tissue was considered day 0 and was retained as normal healthy tissue. Animals were euthanized at 6 and 10 days post-wounding using an overdose of carbon dioxide inhalation.

### Tamoxifen injections

To temporally control Cre-mediated recombination, mice carrying a tamoxifen-inducible Cre variant, *CreERT2*, were crossed to *Ai14* reporter mice (Supplemental Fig. 3)^31^. Using this strategy, injection of tamoxifen was used to determine the temporal expression of Foxd1 in adult palatal tissues. For all studies, a solution of 10 mg/mL tamoxifen in 90% corn oil (Sigma) and 10% ethanol was administered. Tamoxifen injection in postnatal mice was performed on mice heterozygous for both *Foxd1GCE* and *Ai14* alleles. Directly following wounding, mice were subjected to a tamoxifen regimen of 1 mg each day for 3 consecutive days. At 6 and 10 days post wounding, the animals were euthanized, and tissues were collected for histological analyses. Postnatal tamoxifen injection and wounding was performed on 3 animals per timepoint. Tissues from these animals were compared to unwounded palatal tissues, and granulation tissue at 6 and 10 days post wounding from constitutively active *Foxd1GC/Ai14* mice. Tissues from these animals were also compared to unwounded and wounded tissues in *Foxd1GCE/Ai14* that did not receive tamoxifen as a negative control (mice were injected with equal amount of sterile saline).

### Tissue preparation

Isolated embryos were immediately kept in Puck's A Salt Solution after harvesting, fixed in 4% paraformaldehyde at 4 °C overnight, and dehydrated in a sucrose gradient prior to embedding in frozen section compound (Leica). Adult palatal and maxillary tissues were fixed in 10% neutral buffered formalin (Sigma Aldrich, St. Louis, MO) for 24 h at 4 °C and decalcified in 20% EDTA (ethylenediminetetraacetic acid) for 10 days at 4 °C. These tissue samples were then dehydrated in a sucrose gradient prior to embedding in frozen section compound (Leica). Samples were frozen on dry ice and were stored at − 20 °C. Cryosections were cut to 8 μm thickness for subsequent histological analysis.

### Immunohistochemistry and immunofluorescence

Unstained sections to investigate only endogenous fluorescent proteins, were washed in phosphate buffered saline (PBS) and counterstained with Hoechst 33,342 dye (1:1000, Invitrogen, Thermo Fisher Scientific) for nuclei visualization. For antibody labelling, slides were first washed with 1% sodium dodecyl sulfate (SDS) for 5 min, rinsed three times in PBS, and incubated for 30 min in 10% horse serum in PBS for blocking. Tissue sections were then incubated with primary antibodies as shown in Table 1. Primary antibodies were detected using Alexa Fluor IgG secondary antibodies (Invitrogen, Thermo Fisher Scientific). All sections were counterstained with Hoechst 33,342 dye (1:1000, Invitrogen, Thermo Fisher Scientific) for nuclei visualization. Images were taken on Carl Zeiss Imager M2m microscope (Carl Zeiss, Jena) using ZenPro 2012 software. Tissue sections incubated without primary antibodies were used for each antibody labeling to remove background autofluorescence and to confirm specificity of staining.

**Table 1:** Antibodies used for histological assessment.

	Tissue investigated	Antibody ID	Dilution
α-SMA	Adult	Ab5694 (Abcam)	1:100
GFP	Embryonic/Adult	Ab6673 (Abcam)	1:200
PDGFRα	Adult	AF1062 (R&D systems)	1:100
PDGFRβ	Adult	AF1042 (R&D systems)	1:50
CD31	Embryonic/Adult	Ab28364 (Abcam)	1:200
Vimentin	Adult	Ab92547 (Abcam)	1:500
CD146	Embryonic/adult	Ab75769 (Abcam)	1:500
S100β	Adult	Ab52642 (Abcam)	1:500
p75NTR	Embryonic/adult	Ab52987 (Abcam)	1:50
CD105	Embryonic/adult	MAB1320 (R&D systems)	1:100
SOX10	Embryonic	Ab155279 (Abcam)	1:500

### Quantitative histological analysis

For all measurements, only the wounded tissue was quantified, and not the epithelium or the signal from the palatal bone. Quantification of fluorescence staining was performed using macros in ImageJ software to measure the number of tdTomato-positive cells as a ratio to the total number of cells (Hoechst positive nuclei), as well as the percentage of tdTomato-positive cells per field of view (3 different histology sections/ wound, N = 3 animals). For specific cell markers colocalization of the signal of interest with tdTomato-positive cells was manually quantified and presented as ratio of marker-tdTomato-positive cells/total number of tdTomato-positive cells. GraphPad Prism software version 6 (GraphPad, La Jolla, CA) was used to produce graphs. Statistical analyses were performed using 1-way ANOVA or 2-way ANOVA, followed by a Tukey post hoc test as needed. *p* values < 0.05 were considered to be statistically significant.

### Supplementary Information

Supplementary Figure 1.

Supplementary Figure 2.

Supplementary Figure 3.

Supplementary Figure 4.

## Data Availability

The datasets generated during and/or analysed during the current study are available from the corresponding author on reasonable request.

## References

[1] Driskell RR, Watt FM (2015). Understanding fibroblast heterogeneity in the skin. Trends Cell Biol..

[2] Goss G, Rognoni E, Salameti V, Watt FM (2021). Distinct fibroblast lineages give rise to NG2+ pericyte populations in mouse skin development and repair. Front. Cell Dev. Biol..

[3] LeBleu VS, Neilson EG (2020). Origin and functional heterogeneity of fibroblasts. FASEB J..

[4] Hinz B (2016). Myofibroblasts. Exp. Eye Res..

[5] Tomasek JJ, Gabbiani G, Hinz B, Chaponnier C, Brown RA (2002). Myofibroblasts and mechano-regulation of connective tissue remodelling. Nat. Rev. Mol. Cell Biol..

[6] Nikoloudaki G, Snider P, Simmons O, Conway SJ, Hamilton DW (2020). Periostin and matrix stiffness combine to regulate myofibroblast differentiation and fibronectin synthesis during palatal healing. Matrix Biol..

[7] Kalluri R, Weinberg RA (2009). The basics of epithelial-mesenchymal transition. J. Clin. Investig..

[8] Zeisberg EM (2007). Endothelial-to-mesenchymal transition contributes to cardiac fibrosis. Nat. Med..

[9] Crisan M (2008). A perivascular origin for mesenchymal stem cells in multiple human organs. Cell Stem Cell.

[10] Di Carlo SE, Peduto L (2018). The perivascular origin of pathological fibroblasts. J. Clin. Investig..

[11] Hinz B (2010). The myofibroblast: Paradigm for a mechanically active cell. J. Biomech..

[12] Zent J, Guo LW (2018). Signaling mechanisms of myofibroblastic activation: Outside-in and inside-out. Cell Physiol. Biochem..

[13] Humphreys BD (2010). Fate tracing reveals the pericyte and not epithelial origin of myofibroblasts in kidney fibrosis. Am. J. Pathol..

[14] Hung C (2013). Role of lung pericytes and resident fibroblasts in the pathogenesis of pulmonary fibrosis. Am. J. Respir. Crit. Care Med..

[15] Walker JT, Flynn LE, Hamilton DW (2021). Lineage tracing of Foxd1-expressing embryonic progenitors to assess the role of divergent embryonic lineages on adult dermal fibroblast function. FASEB Bioadv..

[16] Gomes RN, Manuel F, Nascimento DS (2021). The bright side of fibroblasts: Molecular signature and regenerative cues in major organs. NPJ Regen. Med..

[17] Chen YT (2011). Platelet-derived growth factor receptor signaling activates pericyte-myofibroblast transition in obstructive and post-ischemic kidney fibrosis. Kidney Int..

[18] Rock JR (2011). Multiple stromal populations contribute to pulmonary fibrosis without evidence for epithelial to mesenchymal transition. Proc. Natl. Acad. Sci. USA.

[19] des Jardins-Park HE, Mascharak S, Chinta MS, Wan DC, Longaker MT (2019). The spectrum of scarring in craniofacial wound repair. Front. Physiol..

[20] Kuhlbrodt K, Herbarth B, Sock E, Hermans-Borgmeyer I, Wegner M (1998). Sox10, a novel transcriptional modulator in glial cells. J. Neurosci..

[21] Yoshida T, Vivatbutsiri P, Morriss-Kay G, Saga Y, Iseki S (2008). Cell lineage in mammalian craniofacial mesenchyme. Mech. Dev..

[22] Grimaldi A, Parada C, Chai Y (2015). A comprehensive study of soft palate development in mice. PLoS ONE.

[23] Byrd KM (2019). Heterogeneity within stratified epithelial stem cell populations maintains the oral mucosa in response to physiological stress. Cell Stem Cell.

[24] Jones KB (2019). Quantitative clonal analysis and single-cell transcriptomics reveal division kinetics, hierarchy, and fate of oral epithelial progenitor cells. Cell Stem Cell.

[25] Yuan X (2019). Wnt-responsive stem cell fates in the oral mucosa. iScience.

[26] Boddupally K, Wang G, Chen Y, Kobielak A (2016). Lgr5 marks neural crest derived multipotent oral stromal stem cells. Stem Cells.

[27] Rinkevich Y (2015). Skin fibrosis. Identification and isolation of a dermal lineage with intrinsic fibrogenic potential. Science.

[28] Broughton KM (2018). Mechanisms of cardiac repair and regeneration. Circ. Res..

[29] Moreira HR, Marques AP (2022). Vascularization in skin wound healing: Where do we stand and where do we go?. Curr. Opin. Biotechnol..

[30] Keswani SG (2013). Role of salivary vascular endothelial growth factor (VEGF) in palatal mucosal wound healing. Wound Repair Regen..

[31] Metzger D, Chambon P (2001). Site- and time-specific gene targeting in the mouse. Methods.

