# Supplementary figures and images for: Assessing the fate and contribution of Foxd1-expressing embryonic precursors and their progeny in palatal development, homeostasis and excisional repair
Source: Sci Rep. 2024 Feb 29;14:4969. doi: 10.1038/s41598-024-55486-8 (PMC10904772; doi:10.1038/s41598-024-55486-8)

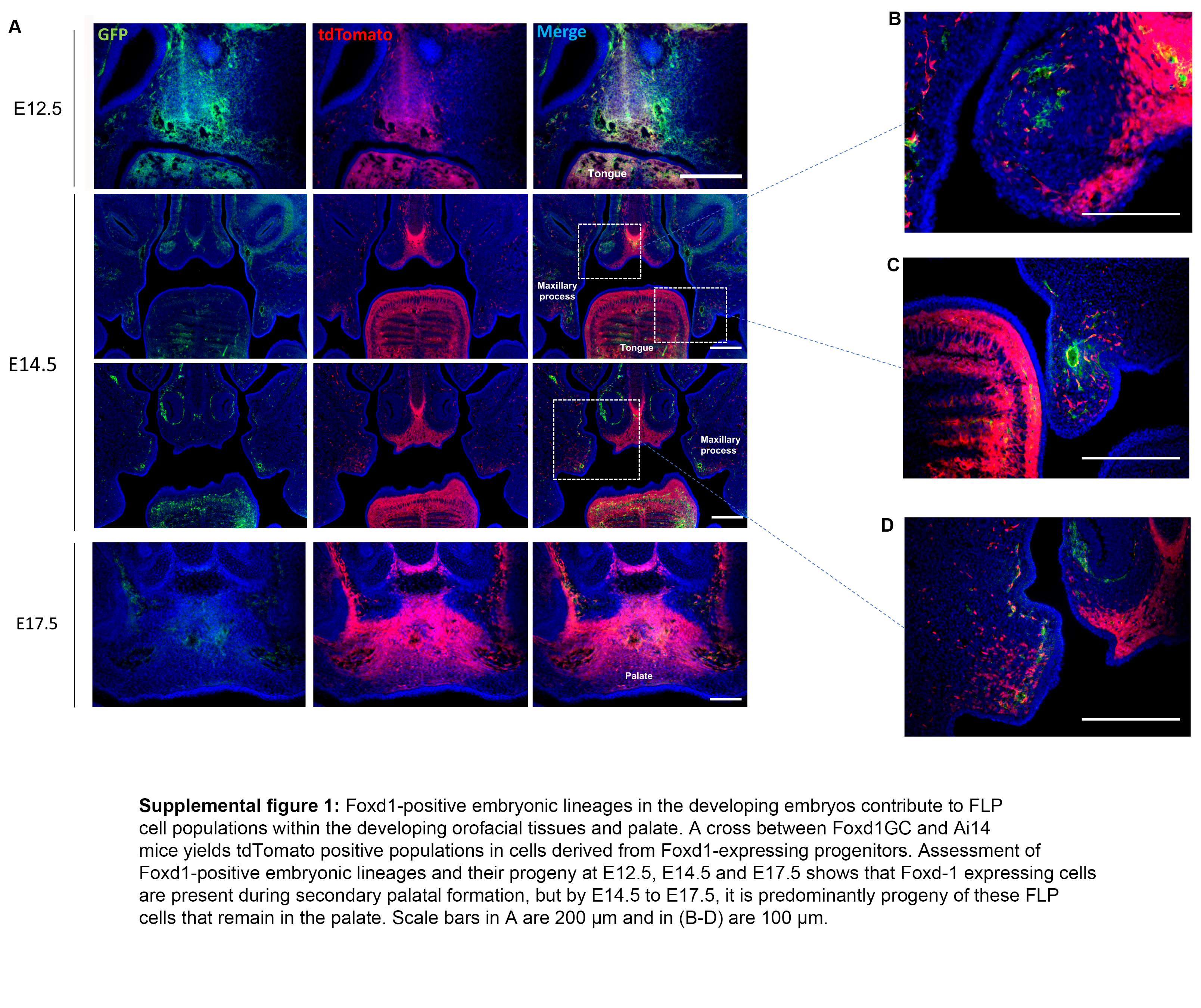

Supplement: Supplementary file 1 — Supplementary Figure 1. [file 41598_2024_55486_MOESM1_ESM.tif]

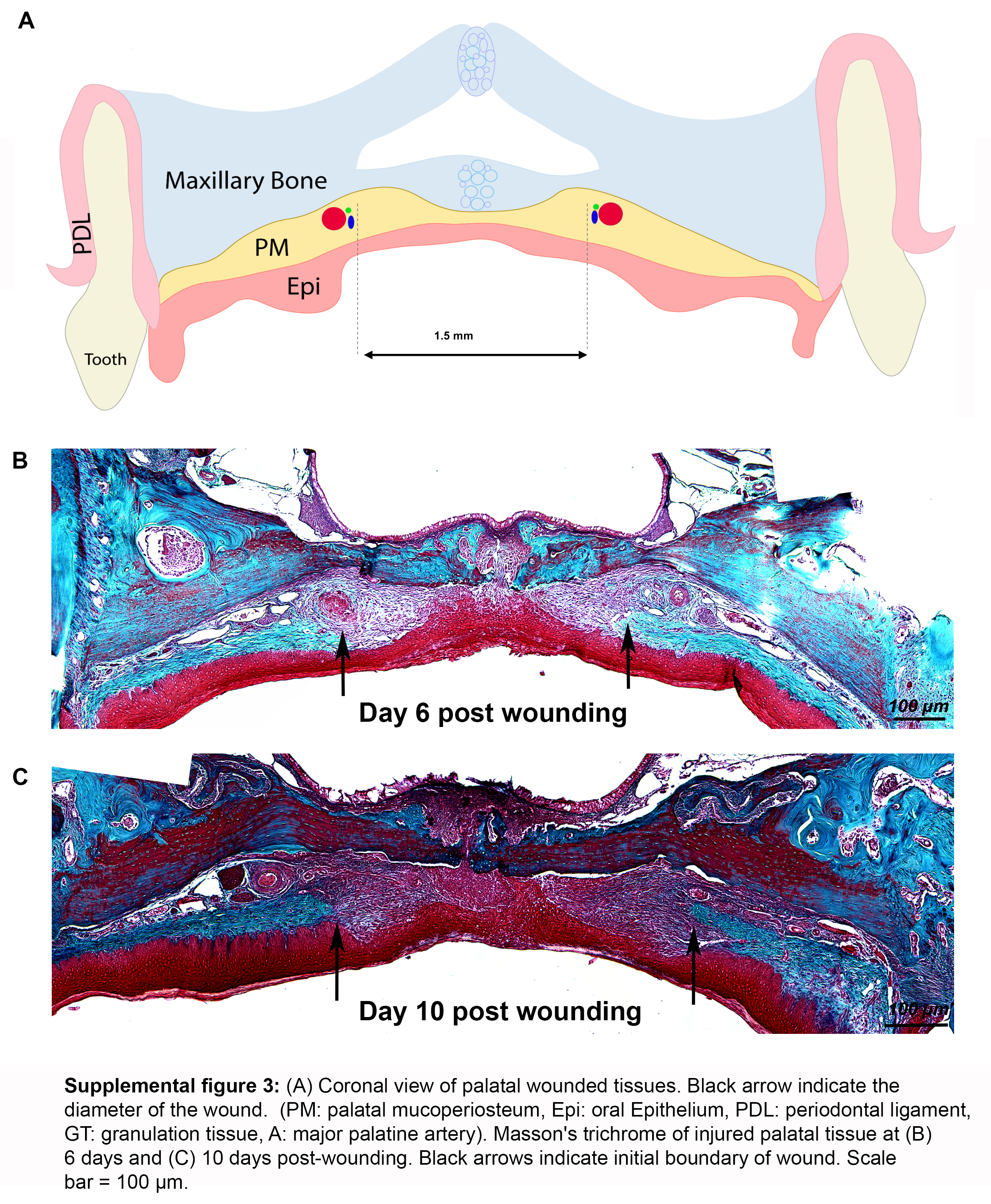

Supplement: Supplementary file 3 — Supplementary Figure 3. [file 41598_2024_55486_MOESM3_ESM.tif]

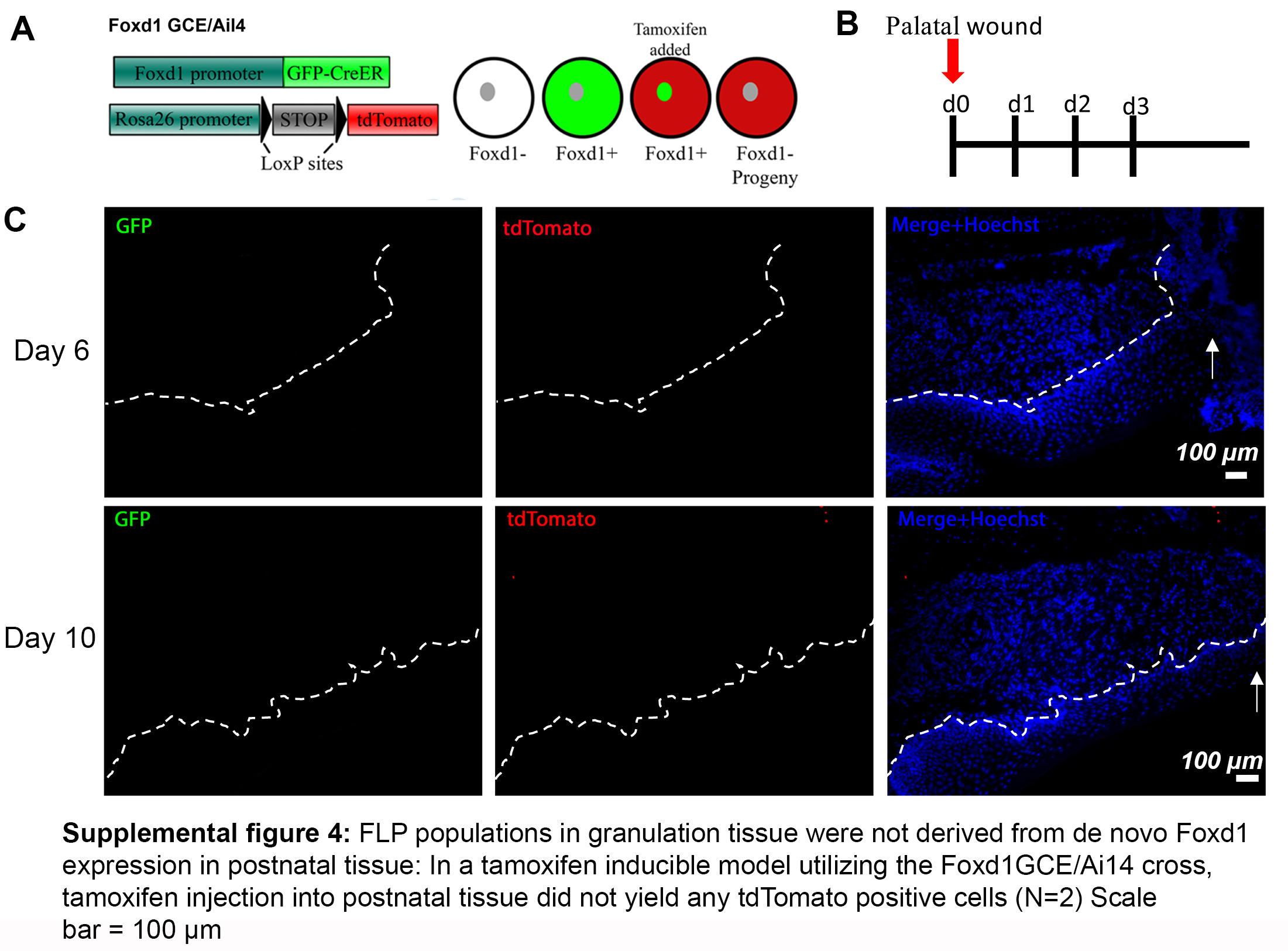

Supplement: Supplementary file 4 — Supplementary Figure 4. [file 41598_2024_55486_MOESM4_ESM.tif]
