# Supplementary material for: Assessing the fate and contribution of Foxd1-expressing embryonic precursors and their progeny in palatal development, homeostasis and excisional repair
Source: Sci Rep. 2024 Feb 29;14:4969. doi: 10.1038/s41598-024-55486-8 (PMC10904772; doi:10.1038/s41598-024-55486-8)

## Slide 1
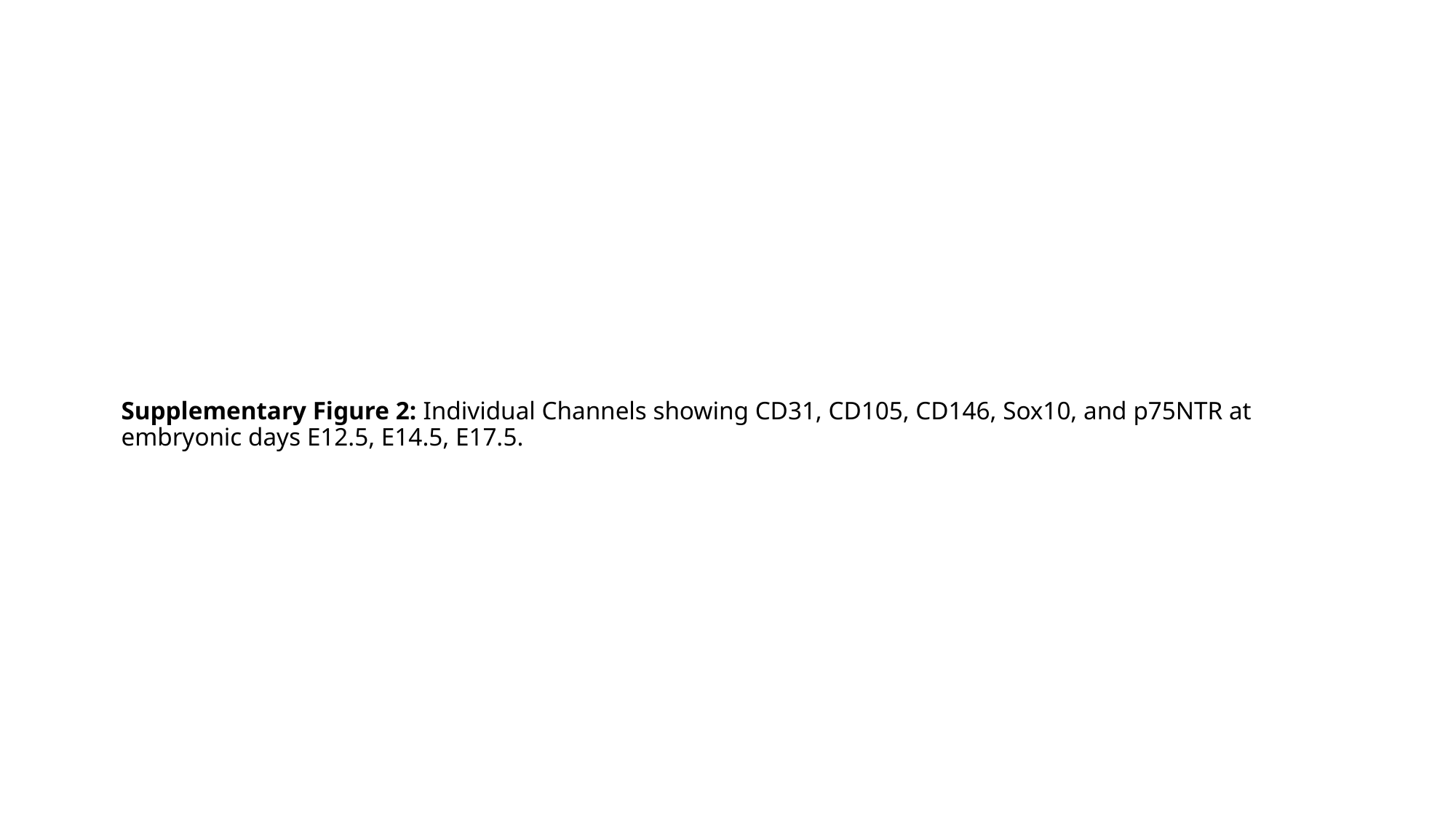

# Supplementary Figure 2: Individual Channels showing CD31, CD105, CD146, Sox10, and p75NTR at embryonic days E12.5, E14.5, E17.5.

## Slide 2
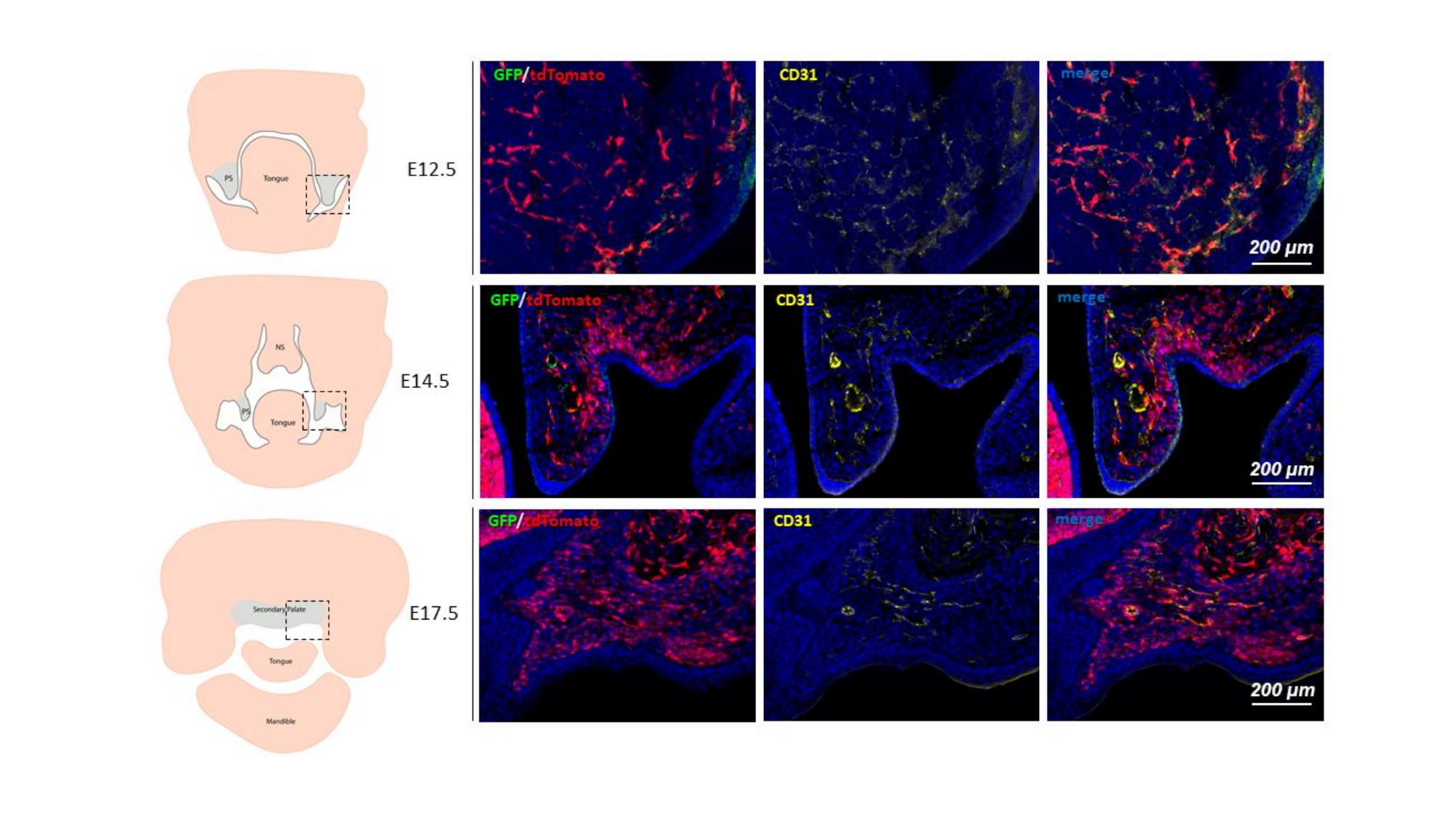

## Slide 3
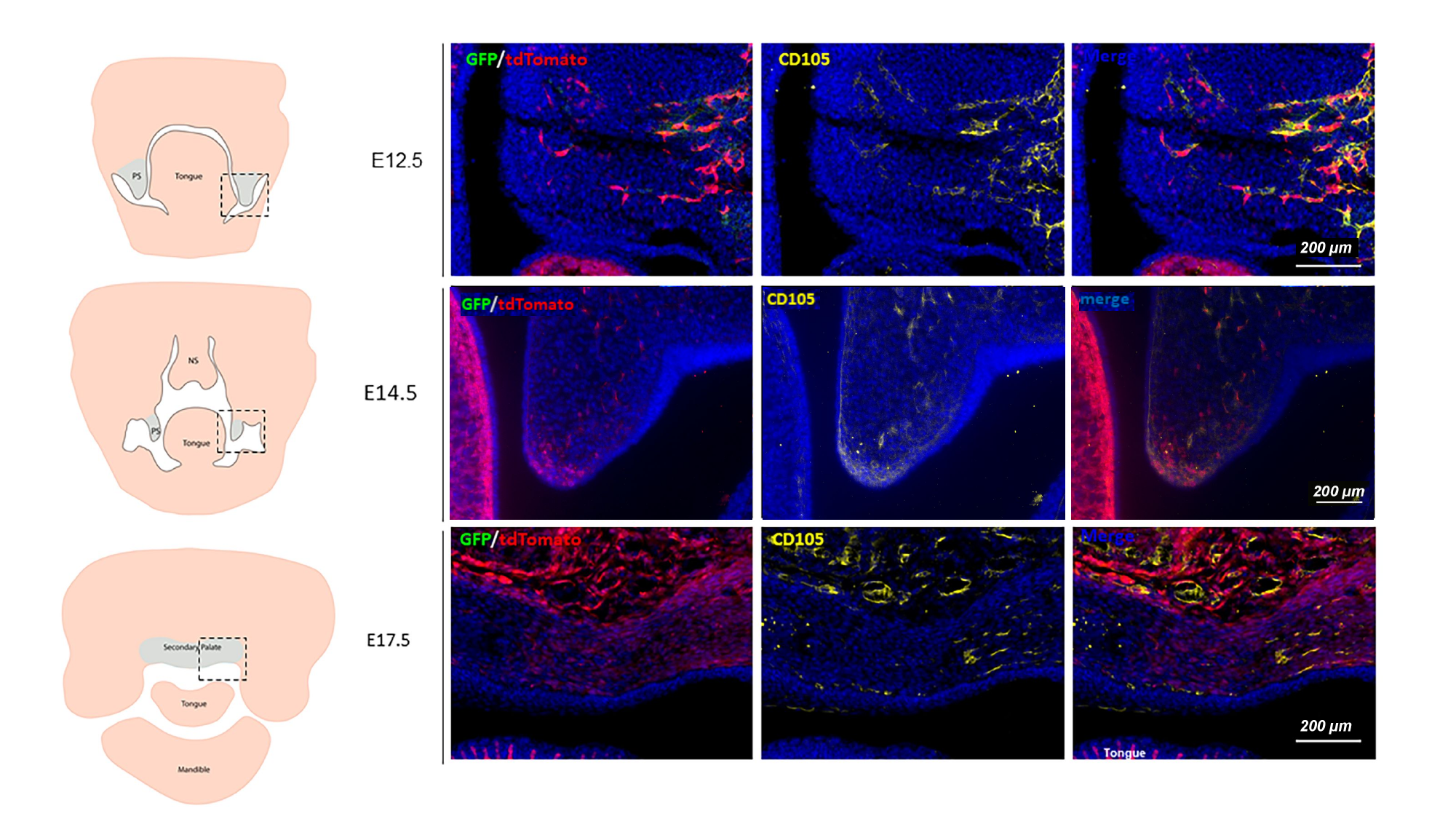

## Slide 4
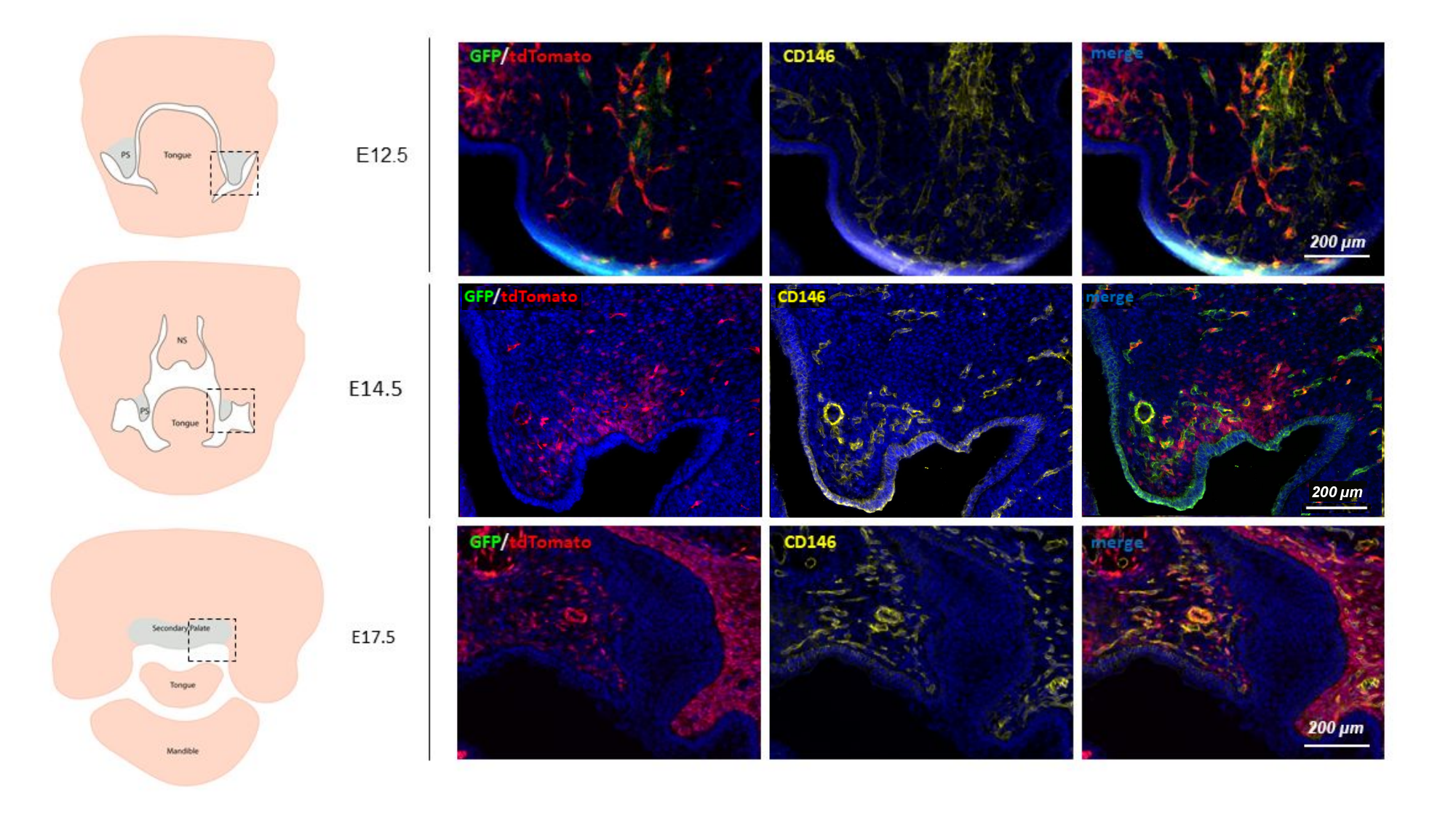

## Slide 5
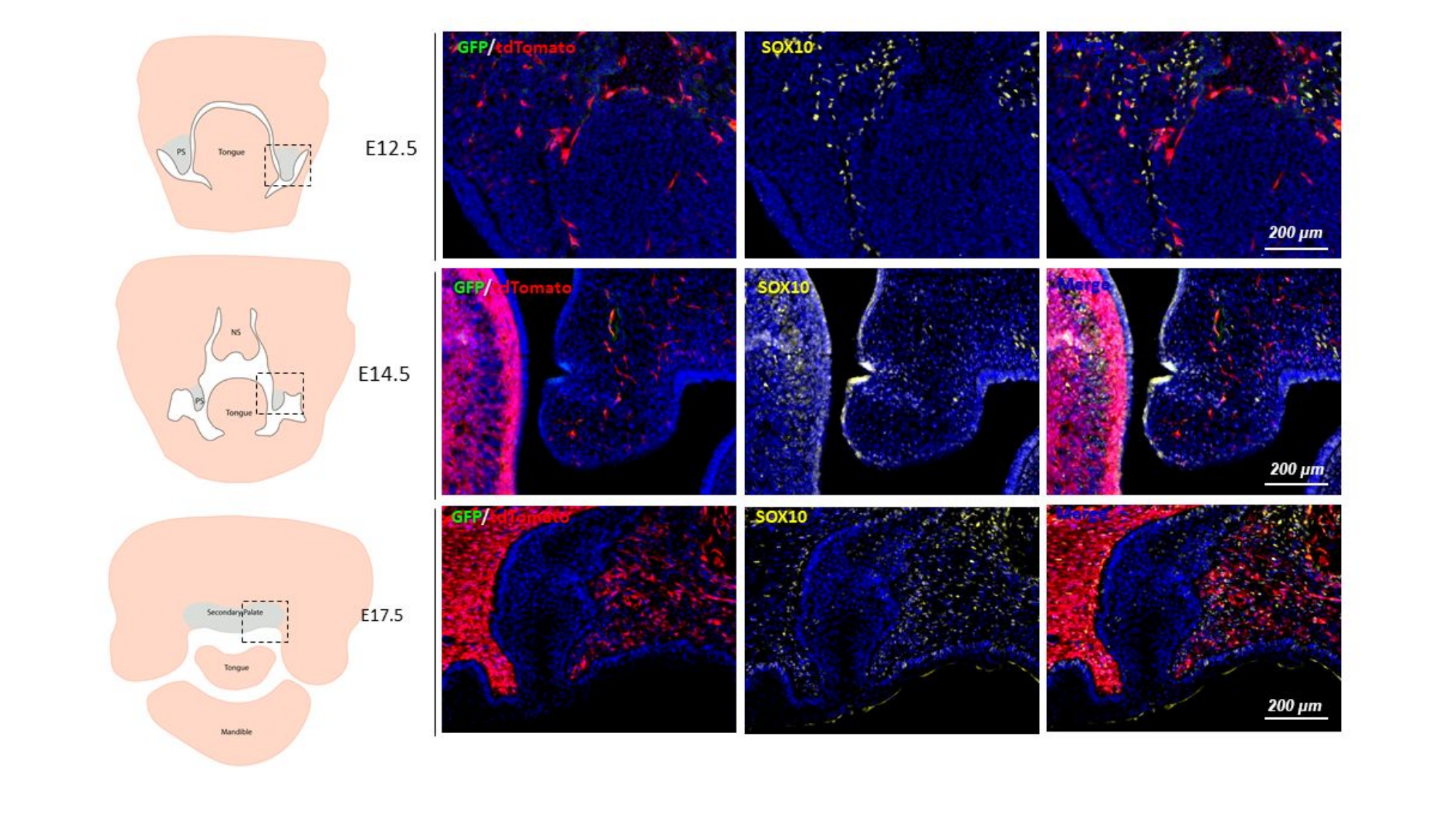

## Slide 6
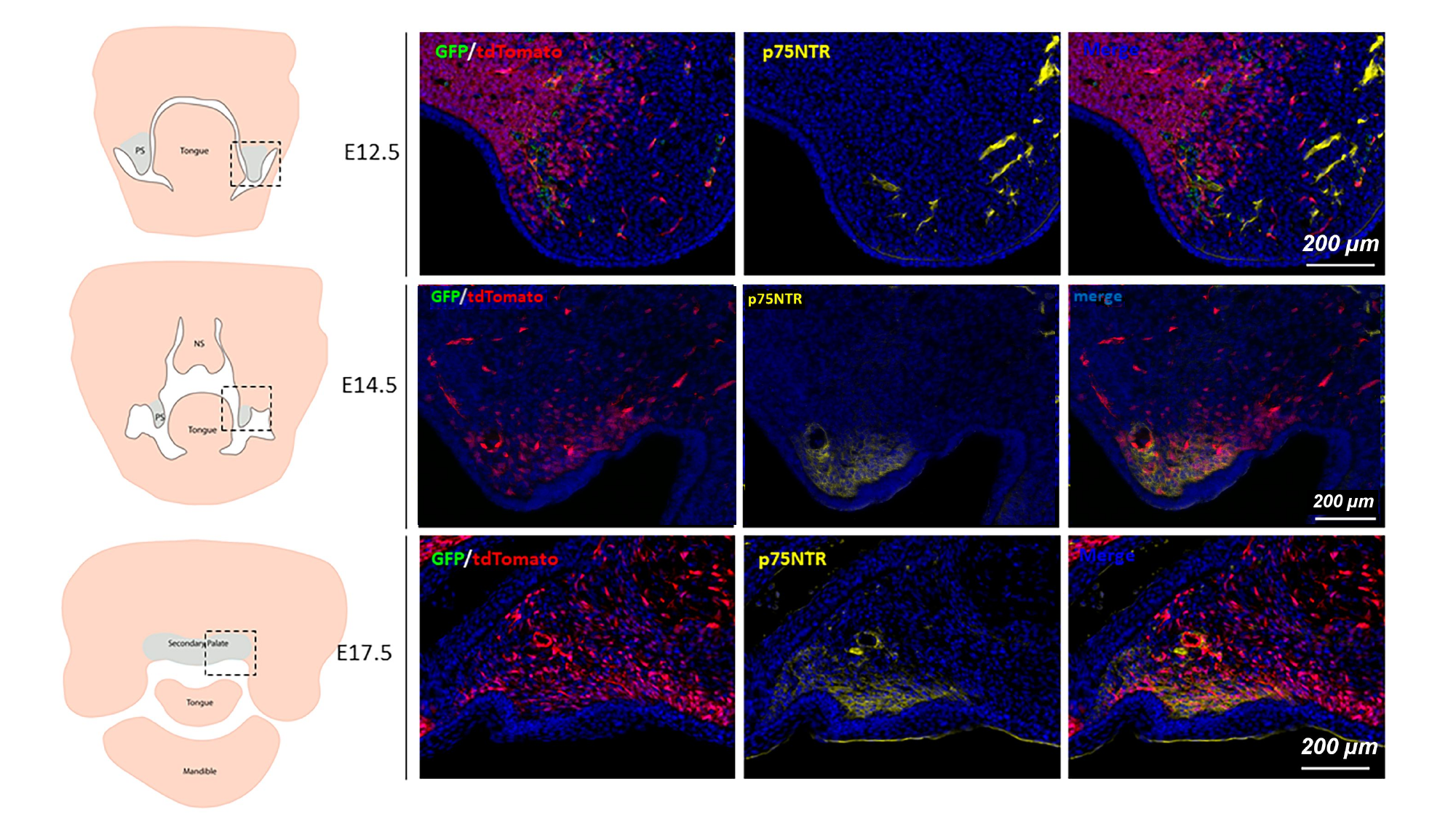

Supplement: Supplementary file 2 — Supplementary Figure 2. [file 41598_2024_55486_MOESM2_ESM.pptx]
